# Umbrella Sampling for Excited States Using a Semiempirical Method

**DOI:** 10.1021/jacsau.6c00302

**Published:** 2026-05-18

**Authors:** Dóra Vörös, Hans Georg Gallmetzer, Johannes C. B. Dietschreit, Sebastian Mai, Leticia González

**Affiliations:** † Institute of Theoretical Chemistry, Faculty of Chemistry, 27258 University of Vienna, Währinger Straße 17, 1090 Vienna, Austria; ¶ Vienna Doctoral School in Physics, 27258 University of Vienna, Boltzmanngasse 5, 1090 Vienna, Austria; § Doctoral School in Chemistry (DoSChem), 27258 University of Vienna, 1090 Vienna, Austria

**Keywords:** nonadiabatic dynamics, umbrella sampling, excited states, photoswitch, conical intersection, semiempirical electronic structure, photoisomerization

## Abstract

Umbrella sampling is widely used to explore ground-state reaction pathways that exhibit high energy barriers and to identify stable molecular conformations. In this work, we extend umbrella sampling to the excited states by using the energy gap between electronic states as a collective variable, not only to locate intersection seams but also to enable the systematic exploration of excited-state relaxation pathways leading to conical intersections. Based on this approach, we present a complete workflow for studying excited-state relaxation mechanisms in detail. As a case study, we apply this method to a push–pull stilbene derivative, 4-(N,N-dimethylamino)-4′-nitrostilbene, using the semiempirical multireference configuration interaction (MRCI) based on the orthogonalization model 2 (OM2) method to simulate photoisomerization. Combining potential energy surface scans, nonadiabatic dynamics simulations, and excited-state umbrella sampling, we identify and characterize five distinct conical intersections for the *trans* and *cis* isomers. Umbrella sampling allows assessing the accessibility of these conical intersections by determining the free energy barriers that separate them from the excited-state minimum. Moreover, it offers insight into the evolution of free energy and entropy along the relaxation pathways, highlighting key thermodynamic features as the system approaches the conical intersections.

## Graphical abstract

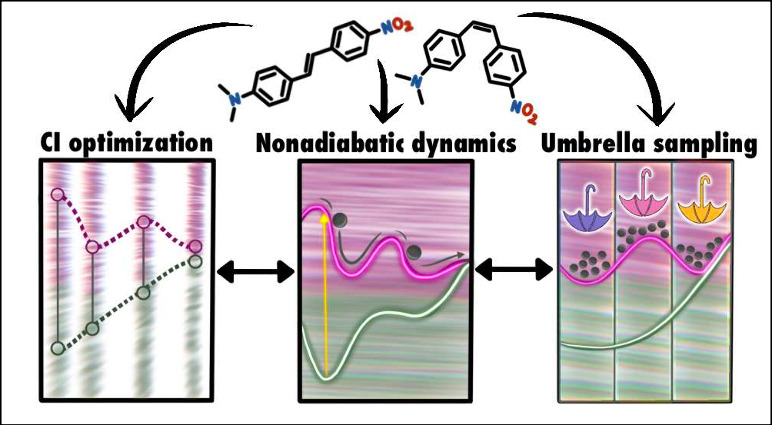

## 1 Introduction

Photochemical reactions have intrigued researchers since the pioneering experiments of Ciamician in the early 20th century.^1,2^ The initial interpretation of photochemical reaction mechanisms was grounded in the noncrossing rule, which posits that adiabatic electronic states of identical symmetry cannot intersect but instead give rise to an avoided crossing—a principle that is strictly valid only for diatomic molecules.^3,4^ In polyatomic molecules, intersections between adiabatic states, known as conical intersections (CIs), can occur, enabling ultrafast, radiationless transitions between different adiabatic electronic states.^5–7^ At these points, the Born–Oppenheimer approximation breaks down because the electronic wave function changes rapidly with the nuclear configuration.

CIs play a crucial role in photochemistry, enabling molecules to access nuclear configurations that are effectively unattainable through thermal activation alone, for example in photoisomerization reactions.^8–11^ Furthermore, CIs provide a radiationless, and often much faster, alternative to radiative deactivation to the ground state, preventing the buildup of long-lived, reactive excited states and thereby ensuring, *e.g.* the photostability of nucleic acids.^12,13^ For these reasons, understanding the structure, energetics, and reaction pathways associated with CIs is essential. Owing to their central role, decades of method development—summarized in Section 2—have been devoted to their identification and characterization. However, most established CI-locating methods have been developed with isolated molecules in gas phase (or within implicit solvent models) in mind, and are not effective for large chromophores or when embedded in explicit environments, such as solvents, biopolymers, proteins, or surfaces. As molecular systems grow in size, individual optimized structures (no matter whether they are global or local minima) become less representative of the full equilibrium ensemble of conformations,^14,15^ making extensive conformational sampling essential for accurately capturing thermodynamic and kinetic behavior. This limitation applies to electronic ground and excited states alike, and thus extends to regions near CIs, where multiple geometries can contribute to the relaxation of a system. In such high-dimensional landscapes, the ruggedness of the potential energy surface (PES) renders individual optimized CIs irrelevant.

In this work, we introduce an excited-state umbrella sampling approach that overcomes the limitations of optimization-based and single-geometry descriptions, allowing comprehensive statistical sampling along a chosen reaction coordinate. This framework is particularly useful for characterizing crossing regions in systems where a single or small set of optimized minimum-energy conical intersections (MECIs) or minimum-energy crossing points (MECPs) is insufficient to capture the relevant nuclear configurations governing nonadiabatic behavior. By enforcing controlled sampling along selected collective coordinates on excited-state PES, excited-state umbrella sampling efficiently explores conformational ensembles in near-degeneracy regions. This ensemble perspective is particularly well suited for large chromophores or those in the presence of explicit environments, where nonadiabatic relaxation is governed by distributions of configurations rather than isolated intersection points. The method is implemented in the open-source SHARC molecular dynamics suite.^16^ We provide a detailed description of the computational protocol, establishing it as a generalizable and robust approach for studying excited state dynamics in near-degeneracy regions. To demonstrate its practical utility, we outline a step-by-step application in 4-(N,N-Dimethylamino)-4′-Nitrostilbene (DANS, see Figure 1), a push–pull photoswitch^17–19^ that can relax radiatively to the electronic ground state via fluorescence or nonradiatively through a CI leading to photoisomerization. Despite extensive experimental characterization in a wide range of solvents^20–28^ and its relevance for numerous applications,^29–33^ the relaxation mechanism of DANS remains incompletely resolved. To address this, we employ umbrella sampling to investigate the relaxation pathway from the excited-state minima toward the CI seam with the electronic ground state, using the energy gap as a reaction coordinate. The simulations are performed using the semiempirical multireference configuration interaction (MRCI) based on the orthogonalization corrected method 2 (OM2),^34–36^ implemented in the MNDO code^37^ and interfaced with SHARC.^16,38^ OM2/MRCI provides the multiconfiguration framework needed to describe *cis*–*trans* isomerizations and the efficiency required to sample the phase space of large, flexible photoactive systems.

**Figure 1:**
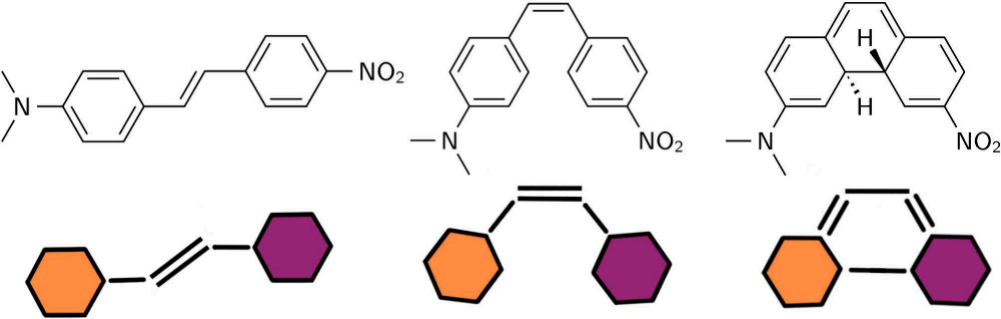
Chemical structures of *trans*-, *cis*-DANS and cyclized form together with the schematic representation. Orange and purple hexagons represent the aromatic rings with the dimethylamino functional group and the nitro group, respectively.

The remainder of this paper is structured as follows. Section 2 provides an overview of existing methods used to explore CIs. Section 3 introduces excited-state umbrella sampling based on the energy gap and the OM2/MRCI method. Section 4 presents the computational details. Results are discussed in Section 5, divided into four parts: static PES scans, nonadiabatic dynamics, analysis of CI geometries, and excited-state umbrella sampling results. Finally, Section 6 concludes this paper.

## 2 Existing Computational Approaches to Find Conical Intersections

Historically, the exploration of photorelaxation mechanisms began with static approaches, in which key stationary points were optimized to map out possible relaxation pathways. As computational tools and algorithms advanced, nonadiabatic excited-state dynamics became feasible, providing not only mechanistic insight but also temporal information on how quickly different relaxation channels are accessed. However, because such dynamical simulations remain prohibitively expensive for large or highly complex systems, static approaches continue to play an essential role to identify dominant and minor relaxation pathways. To overcome their limitations, static methods have evolved into schemes that combine, for example, constrained optimizations with sampling techniques (see below), enabling comprehensive and cost-effective characterization of CIs, including low-probability regions of the seam that are difficult to capture with full dynamical simulations.

When defining CIs, it is important to recognize that they are not isolated points, but rather multidimensional hypersurfaces, commonly referred to as seams, that span an *n* – 2-dimensional space, where *n* = 3*N*_atom_ – 6 is the number of internal degrees of freedom of a nonlinear polyatomic molecule. These hypersurfaces represent regions where the energies of two adiabatic electronic states, labeled as *I* and *J* (where *J* > *I*), become degenerate, fulfilling the condition Δ*E*_*IJ*_ = *E*_*J*_ – *E*_*I*_ = 0. Moving along the two remaining directions lifts the degeneracy of the intersecting electronic states in the first-order approximation, resulting in a cone-like topology—hence the term ‘conical’. The plane formed by these two directions is referred to as the branching plane, *g* – *h* plane or *x*_1_ – *x*_2_ plane, and is orthogonal to the intersection seam.^39–41^ The vectors defining the branching plane are the gradient difference vector

1 $$g_{IJ}=\frac{\partial E_{J}}{\partial R}-\frac{\partial E_{I}}{\partial R}=\frac{\partial }{\partial R}\left(E_{J}-E_{I}\right)$$

which represents the gradient of the energy difference between the two adiabatic states *I* and *J* with respect to the nuclear coordinates $R={\left(R_{1},R_{2},...,R_{3N}\right)}^{T}$ for *N* atoms, and the interstate coupling vector

2 $$h_{IJ}=\left(E_{J}-E_{I}\right)\left\langle \Psi_{I}^{\mathrm{el}}\left|\frac{\partial }{\partial R}\right|\Psi_{J}^{\mathrm{el}}\right\rangle$$

where $\left\langle \Psi_{I}^{\mathrm{el}}\left|\frac{\partial }{\partial R}\right|\Psi_{J}^{\mathrm{el}}\right\rangle =K_{IJ}$ is the nonadiabatic coupling vector with the electronic wave function Ψ^el^.

Over the past three decades, numerous techniques have been developed to identify and characterize CIs. Here, we group these methods into three conceptual “eras”, reflecting the evolving research objectives in exploring CIs. The following overview provides background and context; readers primarily interested in the methodology of the present work may proceed directly to Section 3. Early research focused on the optimization of the MECI, the lowest-energy of the CI seam under the degeneracy constraint.^42–48^ We refer to this as the first era, roughly spanning the 1980s to the early-2000s. Subsequently, it became clear that explaining complex photochemistry requires identifying multiple, local MECIs, prompting the development of new methods capable of exploring beyond a single MECI^49–53^ (we will use the term MECI to denote any local minimum on the CI seam, rather than exclusively the global minimum geometry). We designate this as the second era, spanning the mid-2000s to the mid-2010s, during which it was also recognized that nonadiabatic transitions can occur not only at MECIs but also through a broader region of the CI seam. This motivated the emergence of methods able to systematically explore the wider crossing seam. With advances in computational power and theoretical methods, new objectives emerged, giving rise to sophisticated and automated approaches for sampling CI seams and identifying possible photoproducts following relaxation.^54–57^ We refer to this as the third era, extending from the mid-2010s to the present day, and characterized by methods employing enhanced sampling strategies to stochastically explore the configurational space near the CI seam and dynamically discover relevant intersection regions.

### 2.1 First Era

The first era of CI discovery was driven by the development of methods for optimizing MECIs. These optimization techniques differ in how they guide the system along a vector toward a MECI and can be divided into three categories:^42^ gradient projection, Lagrange multiplier, and penalty function methods. All share the common goal of simultaneously minimizing both the energy difference between electronic states and the total energy of the system.

Gradient projection methods^43,44,58,59^ optimize the geometry by constructing a search vector that closes the energy gap within the *g* – *h* (intersection) space

3 $$f_{1}=2\left(E_{J}-E_{I}\right)\frac{g_{IJ}}{\left|h_{IJ}\right|}$$

and projecting ( $P=I-{\mathrm{g̃}}_{IJ}{\mathrm{g̃}}_{IJ}^{†}-{\mathrm{h̃}}_{IJ}{\mathrm{h̃}}_{IJ}^{†}$ where ${\mathrm{g̃}}_{IJ}$ and ${\mathrm{g̃}}_{IJ}$ denote the orthonormalized vectors) the gradient of the upper state *J* onto the degenerate intersection (sub)­space, which is orthogonal to the *g* – *h* plane (*i.e.*, within the seam space):

4 $$f_{2}=P\frac{\partial E_{J}}{\partial R}$$

The optimization then follows a linear combination of these two gradients **f** = *b*_1_[*b*_2_ **f**_1_ + (1 – *b*_2_)**f**_2_], with different coefficients according to different authors.^43,44,58,59^ The gradient projection method has been integrated with the quantum mechanics/molecular mechanics (QM/MM) framework to enable optimization of local MECI in systems where environmental effects, such as those from solvents or macromolecular surroundings, are included.^60,61^ As noted above, these optimized MECIs might not be representative for such extended systems, but they still helped understand the environmental influence on MECIs better.

Lagrange multiplier methods^45,46,62–67^ combine the energy difference constraints and the coupling condition—defined by setting the off-diagonal element of the electronic Hamiltonian **H** ^el^ to zero—with Lagrange multipliers (ν_1_ and ν_2_), resulting in a Lagrangian function that is optimized within the nuclear coordinate space:

5 $$L_{IJ}\left(R,\nu_{1},\nu_{2}\right)=\frac{E_{I}+E_{J}}{2}+\nu_{1}\left(E_{J}-E_{I}\right)+\nu_{2}H_{IJ}^{\mathrm{el}}$$

with the gradient

6 $$\frac{\partial L_{IJ}\left(R,\nu_{1},\nu_{2}\right)}{\partial R}=\frac{g_{I}+g_{J}}{2}+\nu_{1}g_{IJ}+\nu_{2}h_{IJ}$$

An additional term involving geometric constraint multipliers can be included if specific bond or angle constraints are applied, but that is not considered here. The Lagrange and gradient projection algorithms exhibit comparable performance, but, as demonstrated in ref 42, the Lagrange method might converge more efficiently.

While effective, earlier CI optimization techniques have continued to evolve to improve computational efficiency^68,69^ and to reduce, or even eliminate, the dependence on the computationally demanding interstate coupling vector.^47,48,70,71^ The latter led to the development of a third optimization strategy, known as penalty function methods.^47,48^ These methods introduce a penalty term into the objective function that increases as the energy difference between the electronic states increases. One possible objective function^48^ can be expressed as

7 $$E_{\mathrm{penalty}}=\frac{E_{I}+E_{J}}{2}+\sigma \frac{{\left(E_{J}-E_{I}\right)}^{2}}{\left(E_{J}-E_{I}\right)+\alpha }$$

where σ and α are tunable parameters for the optimization that provide a compromise between accurately locating the MECI and having robust convergence. Although the penalty function method only approximates the CI seam and generally converges less robustly than gradient-projection or Lagrange approaches, it avoids explicit nonadiabatic coupling calculations and thus offers simplicity in the calculation by introducing the tunable parameters.

Despite their effectiveness, all of these methods require a good initial guess of the MECI geometry. This is nontrivial, as MECI structures often differ significantly from molecular geometries near the Franck–Condon region. Moreover, manually selecting the initial guess can introduce bias into the calculation, potentially yielding MECIs that are less relevant to the system’s photochemistry and missing those that actually dominate the relaxation dynamics.

### 2.2 Second Era

The challenges of initial guesses and the limitations of a single MECI spurred the development of methods for identifying multiple MECIs. Although nonadiabatic dynamics approaches can circumvent this limitation, they remain computationally intensive, highlighting the need for efficient static methods to locate multiple MECIs. Moreover, it was shown that nonadiabatic transitions do not occur exclusively at optimized MECIs,^72–76^ underscoring the importance of exploring the wider CI seam region.

To enable a global exploration of MECIs, new techniques were developed by combining the previously introduced optimization methods with strategies to drive the system away from already discovered MECIs toward new ones. The anharmonic downward distortion following method^77,78^ has been used to identify dissociation channels and reaction pathways by following directions where the PES drops more steeply than predicted by the local harmonic approximation. Maeda et al.^49^ later extended the anharmonic downward distortion following approach to multistate crossings, introducing a tailored penalty function that simultaneously minimizes the average energy and the energy gap between two electronic states, thereby enabling automated location of MECIs. This framework was further developed into the seam model function approach, which minimizes a smooth model function combining the average energy and energy gap of two electronic states (similar to penalty function methods), which can also be combined with standard geometry optimization techniques.^50,51^ Additionally, Maeda et al.^79^ combined the seam model function approach with the artificial force-induced reaction method, which systematically explores reaction pathways by applying artificial forces to drive molecular fragments toward reactive configurations and intersection seams.^80,81^

Similar methods have also been developed for MECPs—such as SHAKE-like^82,83^ constrained dynamics simulations^84^ that can aid in addressing intersystem crossing, however, that topic lies beyond the scope of the present work. Furthermore, the nudged elastic band method^85–87^ (traditionally used to identify minimum energy paths on a single PES) and the growing string method (a robust tool for reaction path optimization) have been combined with standard geometry optimization techniques to locate not only MECIs but the crossing seam between two MECIs.^52,53^

Overall, these approaches aim to reduce the arbitrariness of manually selected initial geometries by exploring beyond just the MECI closest to the initial guess. However, even with these developments, comprehensive global exploration of the CI seam remains challenging, particularly for large systems where local search methods might fail to identify all relevant intersections.

### 2.3 Third Era

Since nonadiabatic dynamics can be prohibitively expensive, especially when relaxation is slow or transitions are rare, the third era of CI research is characterized by targeted approaches to sample the CI region efficiently. By generating an ensemble of seam structures, one obtains a more representative picture of the CI region and the pathways that might otherwise be overlooked.

For example, the Crystal algorithm^88^ uses a partitioning of configuration space, similar to a three-dimensional crystal lattice. It eliminates the need for nuclear gradients or collective variables (CVs) by performing simultaneous displacements along multiple degrees of freedom in a discretized configuration space which are evaluated with single-point electronic energies only. New points on the discretized lattice are accepted only if both the energy gap and the average energy of the involved states fall below a predefined threshold. Although the exploration phase is gradient-free, gradient-based techniques are applied afterward to refine the MECI structures. Because the lattice expands fast with system size, the Crystal algorithm becomes quickly computationally expensive for large systems. In such cases, bias-driven searches that steer sampling straight toward zero-gap regions can provide a more efficient alternative for locating multiple MECIs.

Several methods have been developed that deploy enhanced sampling to drive the exploration of the CI seam.^54–57^ Common to them is the use of metadynamics^89–91^ with tailored CVs to drive the exploration of new geometries and the use of an additional biasing term to confine trajectories close to the CI seam. Metadynamics accelerates sampling by periodically adding Gaussian-shaped biases along a selected CV:^89–91^

8 $$E_{\mathrm{MetaD}}\left(t\right)=\underset{t^{\prime }<t}{\sum }w\exp\left(-\frac{{\left(\xi \left(t\right)-\xi \left(t^{\prime }\right)\right)}^{2}}{2\sigma^{2}}\right)$$

where ξ is the CV and *w* and σ the height and width of the Gaussian hill, respectively.

One such algorithm is metaFALCON,^54^ which uses two metadynamics potentials to affect the system’s dynamics. The first potential is directly applied to the ground state PES (the system does not hop) whenever the modified energy gap (Δ*E*_mod_) is large, raising the ground state energy and driving the system toward CI seams. The CV for this metadynamics potential is the modified energy gap

9 $$\Delta E_{\mathrm{mod}}=\sqrt{{\left(E_{J}-E_{I}\right)}^{2}+4E_{g}^{2}}$$

where the energy *E*_*g*_ is the local value of the second metadynamics potential, which depends on a geometric CV and is only applied whenever the modified energy gap is small. This effectively widens the energy gap in regions of the CI seam that have been visited and forces the system to move away to other regions of the same seam or another seam altogether. After the sampling, standard optimization techniques can be applied to refine the sampled MECI structures from the CI seam. Its compatibility with various electronic structure codes makes metaFALCON a highly versatile tool for investigating nonadiabatic processes.

Another method to sample CI seams is the nonadiabatic nanoreactor,^55^ an automated tool designed to explore photochemical reactions without requiring prior knowledge of the photochemistry; it is the nonadiabatic version of the more commonly used ground state nanoreactor for reaction discovery.^92–96^ In contrast to metaFALCON, the dynamics are performed strictly in the excited-state and not on the ground-state PES. A harmonic constraint on the energy gap between excited and ground state pushes the system toward the CI seam, which is then explored using metadynamics with the root-mean-square deviation (RMSD) between aligned molecular structures as a CV, an idea taken from work on conformational exploration.^97^ Beyond simply sampling the CI seam, the nonadiabatic nanoreactor also includes a refinement stage, in which sampled geometries are optimized to a MECI and possible photoproducts are identified by displacing the system randomly along the branching space and optimizing each point on the lower state to locate stationary points. Information about minimum energy paths (barriers) connecting MECIs can also be obtained, as the method employs the growing string method to calculate these paths.

Beyond optimization and sampling techniques, the choice of the underlying electronic structure method is critical, as it is often the computational bottleneck. One way to lighten the computational burden is to employ the extended tight-binding (xTB) method for MECI optimization; here, MECPs are optimized with cost-efficient methods such as GFN2-xTB under the assumption that, in certain systems, the MECPs lie very close to the true MECIs.^56,57^ Similarly to the nonadiabatic nanoreactor, sampling along the seams is enhanced with metadynamics that uses the RMSD as CV^97,98^ and the system is constrained to small energy gaps through an additional bias term. This additional bias is a modified version of the penalty-function (eq 7), where the prefactor σ becomes dependent on the energy gap.

## 3 Methodology

As outlined in Section 2, significant progress has been made in locating CIs and exploring the surrounding hypersurface, highlighting the critical role of CIs in photophysics and photochemistry. Optimizing a single MECI is insufficient to capture the full relaxation mechanism of a molecule. Furthermore, constructing a continuous PES using conventional approaches, such as linear interpolation in internal coordinates^99^ or image-dependent pair potential^100^ methods, becomes impractical for larger systems due to the ruggedness of the PES. Sampling across ensembles of geometries is essential to locate relevant CIs and recover the pathways connecting them. In this work, we employ excited-state umbrella sampling to systematically explore the pathways—and any associated barriers—connecting the Franck–Condon and excited-state minima regions to the CI seam. The following subsections describe our approach in detail.

### 3.1 Excited-State Umbrella Sampling

In addition to the nonadiabatic coupling vector between crossing states, the energy profile leading to the CI is critical. Identifying potential barriers along this path or at the CI itself is important, as such barriers strongly influence reaction probabilities and rates. Therefore, it is desirable to construct an energy profile along a chosen reaction coordinate or CV, ξ­(**R**). This coordinate is usually defined in terms of the nuclear Cartesian coordinates and maps each molecular configuration **R** to its value along the selected CV, enabling the estimation of energy barriers.

Profiles that capture more than just the minimum energy path are the potential of mean force (PMF) and the closely related free energy profile. These reveal not only potential energy barriers but also entropic bottlenecks that can slow down dynamics. To obtain such profiles, one needs to sample configurations from the chosen PES. Since barriers of any kind hamper exploration, a plethora of enhanced sampling methods have been developed, some of which were already mentioned in the discussion of the third era of CI discovery; most of them introduce a biasing potential that modifies the PES. These methods are typically applied to ground-state simulations, but in general there is no restriction on the choice of PES, as long as it is uniquely defined, *e.g.* any adiabatic surface for a fixed electronic state. While the idea of performing adiabatic enhanced sampling in an excited state is not new,^101^ it remains rarely applied due to the computational cost of excited-state simulations.

Some distinctions have to be made when discussing the combination of enhanced sampling with excited states. First, enhanced sampling techniques are used in an adiabatic setting for purely exploratory purposes, to find new configurations with small energy gaps^54^ or new photoproducts.^102^ Second, the sampling is focused along chemically meaningful coordinates to construct PMFs for the adiabatic excited state (as showcased in this tutorial) using explicit excited-state calculations^103^ or force fields fitted to the excited-state PES.^104,105^ Special care has to be taken when the reaction coordinate involves a quantum mechanical degree of freedom, *e.g.* the position of an electron, as this prevents the bias potential and the electronic Hamiltonian from commuting, such that the biasing force must be obtained from a perturbation-like treatment.^101,106^ Third, biasing potentials are used within nonadiabatic simulations to enhance rare surface hops^107^ or to obtain an effective PMF averaged over multiple states.^108^ Fourth, nonadiabatic simulations are combined with path sampling techniques to enhance the occurrence of reactive trajectories, potentially giving access to electronic relaxation rate constants, though these methods typically do not provide profiles. Examples of nonadiabatic transition path sampling include (i) the combination of non-time-reversible fewest-switches surface hopping with transition path sampling,^109^ with the path probability approximately recovered post hoc; (ii) the study of open quantum systems with Lindblad trajectories;^110,111^ (iii) forward flux sampling to obtain rate constants for nonadiabatic events from surface hopping simulations;^112^ and (iv) the very recent combination of transition path sampling with the mapping approach to surface hopping (MASH).^113,114^ Lastly, there exists further literature combining the concept of free energies with excited states that does not fit neatly into the four categories above. Most notable are approaches that apply nonequilibrium free energy corrections using the reference interaction site model self-consistent field method.^115–117^

To reiterate, the use case presented in this paper is the computation of thermodynamic profiles in a single adiabatic electronic state, *i.e.* no nonadiabatic events occur during the simulation. The PMF is obtained by taking the negative logarithm of the marginal probability density ρ­(*z*):

10 $$E_{\mathrm{PMF}}\left(z\right)=-k_{B}T\ln\rho \left(z\right)$$

Here, ρ­(*z*) is the marginal probability density of observing a configuration with ξ­(**R**) = *z*:

11 ρ(z)=∫dR⁡e−E(R)/kBT⁡δ(ξ(R)−z)∫dR⁡e−E(R)/kBT=⟨δ(ξ(R)−z)⟩

where *T* is the absolute temperature, *k*_B_ the Boltzmann constant, and *E* the potential energy.

Different CV choices can yield different probability densities, making the PMF inherently dependent on the transformation of the selected degree of freedom. To obtain a free-energy profile that is independent of the specific coordinate representation, a Jacobian factor must be incorporated when mapping Cartesian coordinates to a specific CV. This factor quantifies how a small volume in the original space is ’stretched’ or ’compressed’ when transforming to the new coordinates, thus reflecting the extent of the Cartesian configuration space associated with each value of the selected CV. To remove the distortion introduced by nonlinear coordinate choices, we employ the set of equations developed by Dietschreit et al.^118^ and implemented in https://github.com/ochsenfeld-lab/adaptive_sampling, where the free energy is calculated using the following expression:

12 $$E_{\mathrm{free}}\left(z\right)=-k_{B}T\ln\left[\rho \left(z\right){\left\langle \lambda_{\xi }\right\rangle }_{z}\right]$$

The brackets with subscript ⟨ ⟩_*z*_ denote an average over the configuration space with the CV fixed at *z*, and λ_ξ_ is the de Broglie thermal wavelength, defined via the sum over the inverse atomic masses (*M*_*a*_) and squared atomic gradients of the CV:

13 $$\lambda_{\xi }=\sqrt{\frac{h^{2}}{2\pi k_{B}Tm_{\xi }}}$$

where

14 $$m_{\xi }^{-1}=\overset{N}{\underset{a=1}{\sum }}\overset{3N}{\underset{i=1}{\sum }}\frac{1}{M_{a}}{\left({\frac{\partial \xi }{\partial R_{ai}}|}_{R}\right)}^{2}$$

The de Broglie thermal wavelength requires the diagonal matrix of the inverse atomic masses **M** ^–1^ and the gradient of the CV $\frac{\partial \xi }{\partial R}$ , quantifying how rapidly the CV changes with respect to the original Cartesian coordinates, thus ensuring that the probability density is correctly weighted in the new coordinate system. The free-energy profile defined in eq 12 can be further decomposed into contributions from the internal energy and entropy along the CV. For the calculation of the internal energy, the potential energy of the respective state on which the sampling is performed, denoted as *E*_α_, is required:

15 $$E_{\mathrm{internal}}\left(z\right)=\frac{3N-1}{2}k_{B}T+\frac{{\left\langle E_{\alpha }m_{\xi }^{-1/2}\right\rangle }_{z}}{{\left\langle m_{\xi }^{-1/2}\right\rangle }_{z}}$$

The entropy can then be determined from the relationship between the free energy and the internal energy:

16 $$S\left(z\right)=\frac{1}{T}\left(E_{\mathrm{internal}}\left(z\right)-E_{\mathrm{free}}\left(z\right)\right)$$

Although this formulation enhances the reliability of the free-energy profile by making it independent of the specific formulation of CVs, caution is still required due to sampling limitations and the challenge of accurately representing the full high-dimensional dynamics with a limited set of CVs.

To construct PMFs or free-energy profiles (plus internal energy and entropy), the key challenge lies in obtaining ρ­(*z*). Our choice to address this is umbrella sampling,^119,120^ which is a method widely used in classical molecular dynamics to sample high-energy regions that are otherwise difficult to thoroughly study by unbiased simulations. To ensure thorough sampling across the entire range of the chosen CV, an additional energy term, known as a bias potential, is applied to focus the sampling of each umbrella window on a specific region of the CV. These simulations are typically performed in overlapping windows along a defined CV, each using a different biasing potential. The probability distributions sampled in each window do not directly reflect the unbiased thermodynamics of the system. To reconstruct the free-energy profile, the data from all windows must be reweighted to recover the unbiased Boltzmann distribution. A common approach to address this challenge is to employ the Weighted Histogram Analysis Method (WHAM)^121–126^ during postprocessing, which combines data from multiple biased sampling windows to reconstruct the unbiased relative energy profile.

The CV serves to reduce complex, high-dimensional molecular motion into a lower-dimensional representation. Therefore, its selection must be made carefully, guided by the specific objectives of the study. In our application, our aim was to investigate the existence of any barrier in the excited state before reaching any CI. For this purpose, we use the energy gap between the intersecting states as the CV:

17 $$\xi \left(R\right)=\Delta E_{IJ}\left(R\right)=E_{J}\left(R\right)-E_{I}\left(R\right)$$

To keep sampling at fixed CV values $\Delta E_{\mathrm{target}}^{i}$ across a set of *W* windows, a biasing energy *E*_bias_ is introduced, represented in the form of a harmonic potential:

18 $$E_{\mathrm{bias}}^{i}\left(R\right)=\frac{k}{2}{\left(\Delta E_{IJ}\left(R\right)-\Delta E_{\mathrm{target}}^{i}\right)}^{2}$$

where Δ*E*_*IJ*_ is the current value of the energy gap, $\Delta E_{\mathrm{target}}^{i}$ is the constant target value of the current window *i*, and *k* is the force constant that determines the strength of the applied harmonic bias. The same biasing energy is added to all the states in the electronic Hamiltonian of the system in order to not alter the gap between the different PESs, but sampling is done in one specific state (*i.e.*, hopping was turned off in the excited-state umbrella sampling simulations). The addition of $E_{\mathrm{bias}}^{i}$ modifies the PES, guiding the system toward configurations that correspond to the target value of the CV, in this case, the energy gap $E_{\mathrm{target}}^{i}$ . The gradients of $E_{\mathrm{bias}}^{i}$ are computed from the forces acting on the specified states *I* and *J* and incorporated into the total force calculations, ensuring that the system remains within the desired window:

19 $$\frac{\partial E_{\mathrm{bias}}^{i}}{\partial R}=k\left(\Delta E_{IJ}-\Delta E_{\mathrm{target}}^{i}\right)\left(\frac{\partial E_{J}}{\partial R}-\frac{\partial E_{I}}{\partial R}\right)$$

Geometric coordinates, like individual bond lengths, angles, or dihedrals each capture only a slice of the nuclear motions that influence energies of states, so picking the ”correct” ones is not straightforward. On the other side, choosing the energy gap Δ*E*_*IJ*_ as the CV means that one does not have to guess which geometric degrees of freedom matter for certain relaxation mechanisms. Here, we note the relation to Marcus theory,^127^ which frames electron transfer processes in terms of two diabatic, harmonic surfaces that are defined with the energy gap Δ*E*_*IJ*_ as the reaction coordinate, since electron transfer can only happen at Δ*E*_*IJ*_ = 0. Therefore, steering simulations along Δ*E*_*IJ*_ guides the system toward the critical crossing geometries while implicitly accounting for the full nuclear reorganization.

The described scheme of performing umbrella sampling in the ground or excited state with Δ*E*_*IJ*_ as the CV has been implemented in the SHARC molecular dynamics program suite.^16^ This implementation is general and can be performed not only along the energy gap (as in the application below) but also using distances, angles, and dihedral angles as CVs (see of the Supporting Information (SI)).

### 3.2 SHARC Dynamics with Semiempirical OM2/MRCI

SHARC (surface hopping including arbitrary couplings)^38,128^ is a general code for excited-state molecular dynamics. In SHARC4.0,^16^ the umbrella sampling is realized through a hybrid interface,^129^ which internally calls one or more other electronic-structure interfaces within the SHARC framework.

Excited-state dynamics impose several requirements on the underlying electronic structure method, including accurate treatment of excited states, availability of energy gradients for nuclear propagation, computation of nonadiabatic couplings or wave function overlaps for state crossings, robust convergent behavior, and sufficient computational efficiency to allow extensive sampling. To perform umbrella sampling toward the crossing seam, efficiency in the electronic structure method is the main bottleneck. An economical alternative to DFT- and wave function-based electronic-structure methods is offered by semiempirical excited-state approaches.^130^ Recently, an interface to the Pisa version of MOPAC (MOPAC-PI^131^) has been made available in SHARC,^132^ to perform semiempirical MRCI based on floating-occupation self-consistent field (SCF) and a variety of semiempirical Hamiltonians (*e.g.*, AM1 ^133^).

As part of the present work, we have interfaced in SHARC the semiempirical MRCI method from the MNDO code developed in Thiel’s group.^34,37,134^ It is based on the OMx Hamiltonians^36,135^ and also supports the ODMx variants,^136^ which include dispersion corrections for improved noncovalent interaction modeling. The OMx method belongs to the neglect-of-diatomic-differential-overlap family of semiempirical electronic Hamiltonians, therefore, three- and four-center integrals are neglected. The one- and two-center integrals are evaluated approximately or parametrized on the basis of experimental data and correlation effects are included in an average manner through the parametrization. Pauli exchange is improved through orthogonalization corrections (to the one-electron integrals in OM1 and to the two-electron integrals in OM2 and OM3).^36,137^ We selected the OM2 Hamiltonian as the most robust and accurate method of the OMx family for our implementation. We decided against using ODM2 because numerical instabilities were observed in the QM/MM framework, resulting in unphysical attractions between MM atoms and the QM boundary. Calculations are based on a minimal valence-only basis set, where core electrons are taken into account through a reduced nuclear charge (assuming complete shielding) and an effective core potential. The molecular orbitals can be computed using standard or floating-occupation molecular orbital^138^ SCF techniques. After the molecular orbitals have been obtained, the electronic states are computed in a second step using MRCI. The MRCI wave functions in the MNDO program can be conveniently defined by specifying (i) a set of reference configuration state functions, (ii) an excitation space covering relevant occupied and virtual orbitals, and (iii) an excitation rank (typically single and double excitations). Within the Graphical Unitary Group Approach (GUGA),^35^ MNDO represents excited states using configuration state functions generated from reference determinants, which are compactly encoded as directed graphs for efficient evaluation of configuration interaction matrix elements.^139^ Due to the small number of reference configuration state functions and a limited excitation space, the number of generated configurations is rather small, which contributes to the high efficiency of the method. However, care must be taken when selecting the reference configurations and excitation space to capture all essential electronic structure features of the states of interest, *e.g.*, by including all valence π/π* and lone pair orbitals. Once all configurations are generated, the MRCI Hamiltonian is diagonalized to obtain excited-state energies and wave functions. Even if it is restricted to singlet states and limited in its provision of parameters (only for H, C, N, O, and F),^36^ due to its efficient analytical gradients and nonadiabatic coupling vectors, the OMx/MNDO framework offers an interesting alternative for excited state studies, including nonadiabatic dynamics simulations.^137^ Example applications include studies of the photostability of DNA nucleobases in different environments (from gas phase to DNA oligomers),^140–146^ mechanisms underlying photoswitches and molecular rotors,^147–151^ or the role of carotenoids in light-harvesting complexes.^152^ More recently, OM2/MRCI has been employed as a reference method for training machine-learning models.^153,154^ Overall, OM2/MRCI provides high efficiency and a reasonable description of excited states,^155^ ideal to efficiently sample excited-state PES.

We note that the nomenclature used in the semiempirical MRCI community differs from that used in wave function-based MRCI implementations. What we referred above as the “excitation space” would, in wave function terminology, be specified by means of frozen core and frozen virtual orbitals, whereas in MNDO for OM2/MRCI simulations is often denoted the “active space”. In the application below, we will not use the term active space to avoid confusion; *i.e.*, MNDO does not perform a full configuration interaction within the active space, nor are active space orbitals in MNDO optimized self-consistently in the correlation treatment. We also want to clarify that in OM2/MRCI in MNDO, only a small number of manually or threshold-selected reference configurations are employed, in contrast to wave function-based MRCI implementations that use sets of references generated from complete active space wave functions.

Our SHARC interface can extract electronic energies, gradients, nonadiabatic coupling vectors, and dipole moments for all electronic states computed with MNDO. Additionally, the interface can compute wave function overlaps based on molecular orbital and configuration interaction coefficients (note that no atomic orbital overlaps are needed within the MNDO approximation) through the WFOverlap program.^156^ These overlaps are used instead of the more expensive nonadiabatic coupling vectors to propagate the electronic wave function.^157,158^ Moreover, the interface is able to handle electrostatic embedding for QM/MM calculations.

### 3.3 Workflow

Figure 2 summarizes the SHARC workflows for nonadiabatic dynamics and excited-state umbrella sampling using OM2/MNDO. The flowchart is organized into three columns corresponding to the main computational layers: (i) the general simulation management (“Project workflow”), (ii) the dynamics driver responsible for propagating a single trajectory (“Trajectory workflow”), and (iii) the electronic-structure calculation executed through the SHARC interface infrastructure (“Interface workflow”).

**Figure 2:**
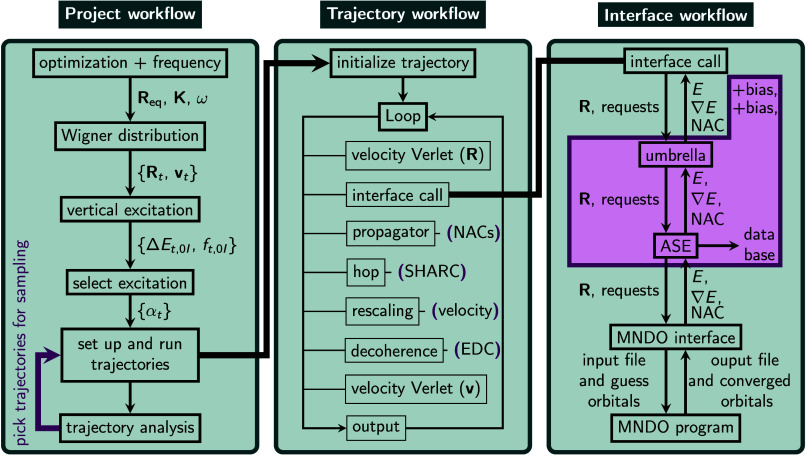
Workflow of SHARC divided into three panels: “Project workflow”, “Trajectory workflow”, and “Interface workflow”. Black arrows indicate connections between panels, and purple-colored sections highlight elements specific to umbrella sampling.

The first step in a gas-phase dynamical simulation is the preparation of initial conditions. Here, we use Wigner sampling for the unbiased nonadiabatic dynamics. For that, SHARC uses an optimized equilibrium geometry (**R**_eq_), normal mode vectors (**K**), and vibrational frequencies ω from these calculations to generate an ensemble of initial geometries (**R**_*t*_) and velocities (**v**_*t*_). Subsequently, vertical excitation calculations are performed on each generated geometry to obtain excitation energies Δ*E*_*t*,0*I*_ and oscillator strengths *f*_*t*,0*I*_. An energy window or a specific excited state can then be defined, from which initial conditions with the active state α_*t*_ for the dynamics are selected stochastically. With this information, the trajectories can be set up and initialized. After reaching the required time step, the SHARC analysis tools are available to obtain information from the simulation, concluding the project workflow.

While running a set of trajectories, each is propagated independently by the SHARC dynamics driver. Each iteration begins by propagating nuclear positions using the velocity Verlet algorithm. An external electronic structure interface (described in the next paragraph) is then invoked to compute energies, gradients, and nonadiabatic couplings. The electronic wave function is propagated as

20 $$\frac{\partial }{\partial t}c_{I}\left(t\right)=-\underset{J}{\sum }\left[\frac{i}{\hbar }H_{IJ}^{\mathrm{el}}+T_{IJ}\right]c_{J}\left(t\right)$$

where *c*_*k*_ are the coefficients of the electronic wave function (in this work, there are three coefficients for the three singlet states) and **H** ^el^ is the electronic Hamiltonian. The time-derivative coupling $T_{IJ}=\left\langle \Psi_{I}^{\mathrm{el}}\left|\frac{\partial }{\partial t}\right|\Psi_{J}^{\mathrm{el}}\right\rangle$ relates directly to nuclear motion through the nonadiabatic coupling vector **K**_*IJ*_ as *T*_*IJ*_ = **v** ·**K**_*IJ*_. The surface hopping probabilities are computed using the standard SHARC hopping probabilities,^159^ including decoherence correction and velocity rescaling as needed. Finally, velocities are updated via the velocity Verlet integrator, and the loop continues.

The interface call step is responsible for communication between SHARC and the external electronic structure method at every step of the trajectory loop. Here, the dynamics engine invokes the MNDO interface, which prepares the necessary input files and initial guess orbitals for the MNDO calculations. After MNDO completes the electronic structure calculation, the interface collects the output data and updated orbitals, then returns the requested properties (energies, gradients, and couplings) back to SHARC for the next propagation step.

The parts of the workflow related to umbrella sampling are highlighted in purple. To set up umbrella sampling, geometries and initial velocities were selected from previously propagated trajectories, picking time steps with specific energy gap values or dihedral angles. In the trajectory workflow, purple brackets indicate settings irrelevant for umbrella sampling (electronic wave function propagation, hopping, rescaling, decoherence), as surface hopping is disabled in this step. The additional umbrella sampling-specific step in the interface workflow is highlighted with a purple-colored frame. This includes the umbrella interface, which applies biases to the energies and gradients before returning them to SHARC for propagation. Furthermore, an additional hybrid interface above the MNDO interface is invoked, which writes out the unbiased energies and gradients into a database using the atomic simulation environment (ASE) package.^160^ Positioning the ASE interface above the umbrella interface would instead save biased energies and gradients, showing the flexibility of a modular computational workflow.^129^ For instance, extending these calculations to a QM/MM framework could be easily achieved by inserting a QM/MM hybrid interface between the MNDO and ASE interfaces. In this case, the QM/MM hybrid interface would call the MNDO interface and an additional MM engine and ASE would store QM/MM energies and gradients before the umbrella sampling bias is added. A more detailed guide to the umbrella sampling procedure is provided in of the SI.

## 4 Computational Details

To showcase the scope of excited-state umbrella sampling, we study the photochemical behavior of DANS in the gas phase, combining static characterization of the relaxation pathways, nonadiabatic dynamics simulations, and excited-state umbrella sampling with the energy gap as the CV. Below we describe the computational details of each part separately.

### 4.1 Level of Theory of the Electronic Structure Calculations

Electronic structure calculations are done with the OM2/MRCI method implemented in the MNDO package^34–37^ (version MNDO2020). First, molecular orbitals are obtained via restricted open-shell Hartree–Fock, using the minimal valence-only basis set that belongs to the OM2 method. Then, for the actual OM2/MRCI calculation, we use an excitation space consisting of four electrons in four orbitals, comprising the two highest lying π orbitals and the two lowest lying π* orbitals (HOMO–1, HOMO, LUMO, LUMO+1, see ). The HOMO is localized on the central C = C bridge and the NMe_2_ substituted ring, whereas and the LUMO is predominantly localized on the NO_2_ substituted ring. This excitation space yields an adequate description of the S_1_, which is mostly characterized by a HOMO → LUMO transition and dominates the absorption spectrum.^26,161–164^ We note that this excitation space does not correctly capture the dark S_2_ (nπ*) state; however, expanding the excitation space with the required NO_2_-centered lone pairs led to bad convergence behavior and total energy conservation issues. For further details on the choice of excitation space see in SI. Thus, the four-orbital excitation space is employed as an optimal balance between the accurate description of S_1_ and the computational robustness of the dynamics. To benchmark the chosen excitation space, we performed additional calculations and compared the results with previously published TD-DFT data.^164^ As shown in in the SI, OM2/MRCI reproduces the TD-DFT results well for both the *cis* and *trans* isomers, with root-mean-square deviations of 0.17 and 0.18 eV, respectively.

Within the four-orbital excitation space, we employed six reference configurations plus all single and double excitations from these references. This setup covers all 12 singlet configuration state functions that exist for a (4,4) complete active space. Thus, our electronic structure level of theory could be characterized as a semiempirical CASCI­(4,4), based on restricted-open shell self-consistent field orbitals.^165–167^

### 4.2 Potential Energy Scans

As a first step to understand the relaxation mechanism of DANS from the S_1_ state, we optimized the S_0_ minimum, the S_1_ minimum, S_1_/S_0_ MECIs, and a transition state (TS) between the S_1_ minimum and S_1_/S_0_ MECIs. Subsequently, we connected these optimized geometries by scans. All steps of this procedure were performed the same way for both *trans* and *cis* isomers.

The S_1_/S_0_ MECIs and the S_1_, S_0_ minima of both isomers were optimized using the SHARC program package, feeding the ORCA^168^ geometry optimizer engine with energies and gradients from MNDO, explained above. The gradient to follow was constructed using a gradient projection method combined with efficient branching plane update.^48^ To locate the TS in the S_1_ state between the S_1_ minimum and the S_1_/S_0_ CIs, first nudged elastic band calculations^85–87^ were performed with 24 images using the default spring constant that varies between 0.01 and 0.10 Hartree/Bohr, followed by a TS optimization refinement. To obtain a continuous relaxation path and verify whether there is an additional barrier between the optimized points, we interpolated between the S_0_ and S_1_ minimum via the image-dependent pair potential method^100^ as implemented in ORCA. For the segments S_1_ minimum → TS → MECI­(S_1_/S_0_), the minimum energy pathways obtained from the nudged elastic band calculations were used. For the segments where the nudged elastic band calculations and transition-state optimizations did not converge, the highest-energy point from these nudged elastic band calculation was taken, and interpolation between the points was performed using the image-dependent pair potential method.

### 4.3 Nonadiabatic Dynamics

While PES scans provide qualitative insight into competing relaxation pathways, quantitative photophysical kinetic information requires nonadiabatic dynamics simulations. In addition to rate constants, dynamical simulations can access additional CI geometries not identified in static optimizations. The resulting structures serve as valuable starting points for discovering additional CI geometries, which were overlooked in static optimizations, that can then be refined and added to the set of local MECIs.

The nonadiabatic dynamics simulations were performed using the SHARC program package,^16,38^ at the OM2/MRCI level of theory. For each of the two isomers, 1000 initial conditions were sampled from a Wigner distribution using the ground-state normal modes obtained from a frequency calculation. Two excited singlet states were calculated for each geometry and the resulting excitation energies and oscillator strengths were used to generate an ensemble-broadened absorption spectrum.^169–171^ Despite the limitations in describing the S_2_ state, for the nonadiabatic dynamics the S_2_ state is included to serve as a buffer state above the targeted S_1_ state. Subsequently, 200 out of 1000 initial conditions were stochastically selected from the energy window of 3.2 to 4.0 eV, centered on the first absorption band of each isomer to selectively excite to the first excited state (). The sets of 200 trajectories were propagated for 20 ps for the *trans* isomer and 5 ps for the *cis* isomer, considering the ground state and two excited states. The nuclei were propagated with a time step of 0.1 fs (which implies 200,000 electronic structure calls for the *trans* and 50,000 for *cis* simulations), while the electronic equation of motion was solved with 25 substeps (0.004 fs per substep). For the electronic propagation, analytical nonadiabatic couplings were used, since MNDO does not provide phase-consistent wave function overlaps. To conserve the total energy after a hop, the velocity vector was rescaled in the direction of the nonadiabatic couplings. The energy-based decoherence correction scheme of Granucci and Persico^172^ was applied with the standard parameter of 0.1 au.

Because previous applications of OM2/MRCI have shown that spikes in the gradients can occur,^173^ leading to an increase in the total energy during dynamics, we carefully monitored the total energy throughout the simulations. Trajectories that failed to conserve the total energy before hopping to the ground state were discarded to avoid artificial transitions to the ground state caused by sudden changes in gradients and energies; see examples in . Accordingly, from the 200 trajectories, we kept 128 for the *trans* and 192 for the *cis* isomer. This large difference in the number of crashed trajectories likely results from the longer simulation time and greater flexibility of *trans*-DANS.

### 4.4 Umbrella Sampling

Excited-state umbrella sampling not only offers an alternative approach to obtain potential energy scans but enables the estimation of energy barriers along the pathway to the CI seam. Umbrella sampling was performed adiabatically in the S_1_ state without any population transfer to other electronic states using the S_1_/S_0_ energy gap as CV using the SHARC program package at OM2/MRCI level of theory. Sampling windows were placed at 0.1 eV energy gap intervals, ranging from 3 to 0 eV. These closely spaced windows were selected to ensure sufficient overlap between the windows. The force constant of eq 18 was set to 4.6 eV^–1^, based on preliminary test runs aimed at balancing efficient confinement within each energy gap region with sufficient overlap between adjacent windows. Automated tools can also be used to select suitable umbrella sampling parameters.^174^ The initial geometries for sampling were obtained from prior unbiased nonadiabatic dynamics simulations, where structures with the desired energy gap were identified. Alternatively, sampling can be started from geometries outside the desired energy gap region, although equilibration around the defined constraints is first required in such cases. Each umbrella sampling simulation was conducted for 5 ps in the S_1_ state using the same setup as described in Section 4.3, but disabling hopping to the ground state (thus, the choice of wave function propagation, decoherence, and velocity vector rescaling becomes irrelevant for the sampling). Geometries were collected from the trajectories at 2 fs intervals, and for each geometry, the S_1_ energy, energy gap S_1_/S_0_ and forces of S_1_, S_0_ states were extracted.

In addition to the energy gap-based umbrella sampling, a separate set of simulations was carried out using the central dihedral angle (HCCH)—involving the bridging moiety between the two aromatic rings—as the CV. These simulations used the same setup and a force constant of 106 kcal mol^–1^ rad^–2^. The windows were set in 3° spacing from 180° to 90° for *trans* and from 0° to 90° for the *cis* isomer.

Calculation of free energy, internal energy, and entropy was performed using the method developed by Dietschreit et al.,^118^ as outlined in Section 3.

## 5 Results and Discussion

### 5.1 Static Scan of Excited-State Relaxation Pathways

The possible relaxation pathways of *trans*- and *cis*-DANS starting from the S_1_ state are summarized in Figure 3. These pathways have been found by minimum energy scans performed along relevant reaction coordinates based on literature for stilbene or on results from our dynamics simulations or umbrella sampling. The S_1_ state corresponds to a bright charge-transfer excitation from the dimethylamino group and its adjacent ring to the nitro group and the ring to which it is attached.^164,175–177^ Energies and oscillator strengths of the optimized geometries are collected in , barrier heights and energy gaps between S_1_ and S_0_ at the TS are in (both in ).

**Figure 3:**
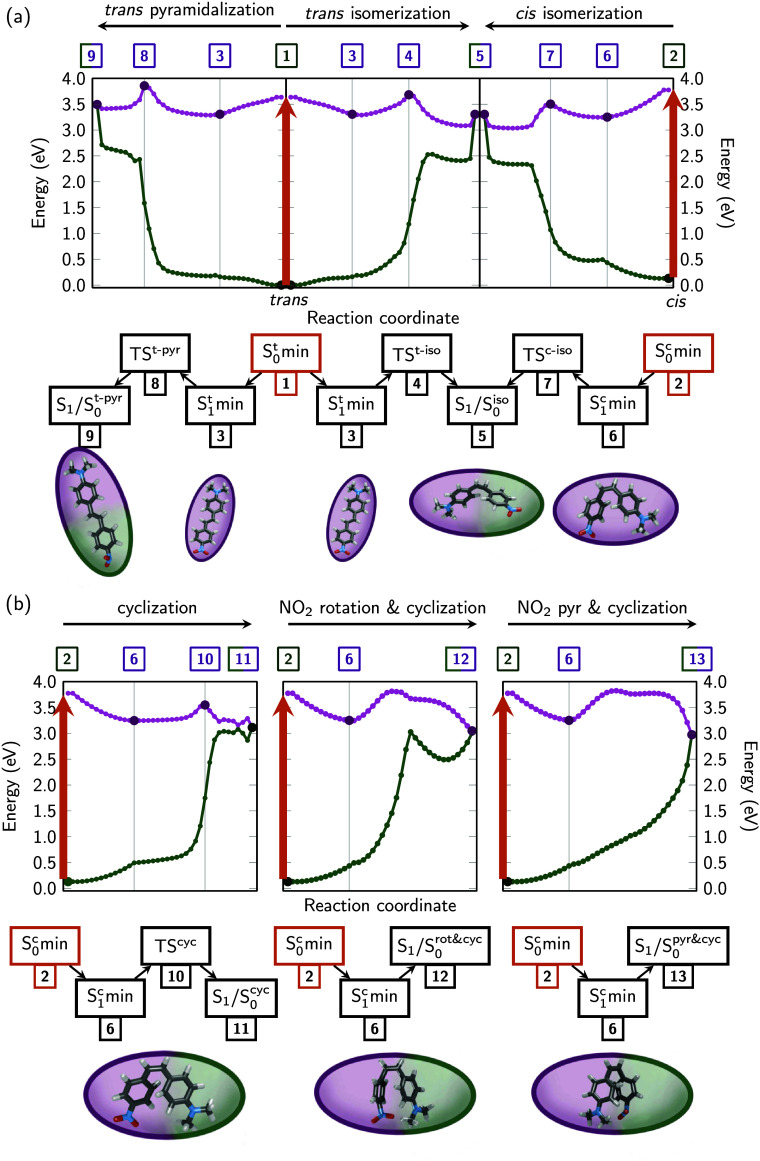
Relaxation pathways of *trans*- and *cis*-DANS optimized at the OM2/MRCI level of theory. Critical points are marked by vertical light-gray lines and numbers above. S_1_ and S_0_ energies are in purple and green, respectively. Orange (arrow and rectangles) denotes excitation at the Franck–Condon region. Local S_1_ minima and MECIs are labeled as S_1_/ $S_{0}^{\mathrm{pyr}}$ , $S_{1}^{t}$ min, S_1_/ $S_{0}^{\mathrm{iso}}$ , $S_{1}^{c}$ min, S_1_/ $S_{0}^{\mathrm{cyc}}$ , S_1_/ $S_{0}^{\mathrm{rot\&cyc}}$ , and S_1_/ $S_{0}^{\mathrm{pyr\&cyc}}$ , with superscripts “t” and “c” indicating *trans* and *cis*, “pyr” for pyramidalization, “cyc” for cyclization, “rot” for rotation and “iso” for isomerization pathways.

Using initial guess geometries known in the literature for stilbene,^178–180^ we could identify the S_1_/ $S_{0}^{\mathrm{iso}}$ and S_1_/ $S_{0}^{\mathrm{cyc}}$ CIs, corresponding to the isomerization and cyclization pathways, respectively. We discovered the S_1_/ $S_{0}^{\mathrm{pyr}}$ , S_1_/ $S_{0}^{\mathrm{rot\&cyc}}$ and S_1_/ $S_{0}^{\mathrm{pyr\&cyc}}$ CIs through nonadiabatic dynamics and excited-state umbrella sampling, illustrating the importance of including dynamical procedures to obtain a complete relaxation landscape.

The orange vertical arrows indicate excitation to the S_1_ state from the respective Franck–Condon region of each isomer. The main relaxation pathway for *trans*-DANS corresponds to the sequence 1 → 3 → 4 → 5, which represents the isomerization route. Relaxation to the S_1_ minimum ( $S_{1}^{t}$ min) is followed by a 0.33 eV barrier toward the S_1_/ $S_{0}^{\mathrm{iso}}$ MECI leading to isomerization. The $S_{1}^{t}$ min structure is planar and involves rotation around the central C–C bond to reach the S_1_/ $S_{0}^{\mathrm{iso}}$ degeneracy. This particular MECI is known in the literature for DANS^181–183^ as the “perpendicular” geometry, since the HCCH dihedral angle at the carbon bridge is approximately 90°. From there, the molecule can relax to the ground state, leading either to the *cis* isomer or back to the initial *trans* isomer.

A similar isomerization pathway is found for *cis*-DANS. Starting from the second orange arrow (right in Figure 3a), and following the path 2 → 6 → 7 → 5, the system can also reach this “perpendicular” S_1_/ $S_{0}^{\mathrm{iso}}$ MECI. For the *cis* isomer, the pathway from the equilibrium geometry ( $S_{0}^{c}$ min) to the $S_{1}^{c}$ min is steeper, suggesting that more excess energy is available to overcome the barrier separating the minimum from the S_1_/ $S_{0}^{\mathrm{iso}}$ . The barrier itself is slightly lower (0.28 eV) than in the *trans* isomer, indicating a faster relaxation to S_1_/ $S_{0}^{\mathrm{iso}}$ when the molecule is excited from the *cis* configuration.

In addition to the pathways that are also known for stilbene,^178–180^ three alternative routes were identified from nonadiabatic dynamics simulations and the excited-state umbrella sampling described below. In this case, the hopping geometries are optimized as MECIs and used to calculate the minimum energy pathways to the corresponding S_1_ minima. For *trans*-DANS, the pathway 1 → 3 → 8 → 9 involves twisting and pyramidalization of the nitro group. This MECI (S_0_/ $S_{1}^{t‐\mathrm{pyr}}$ )—associated with NO_2_ pyramidalization—was not reported before, although experimental evidence pointed to the existence of a CI involving NO_2_ vibrational modes.^19,21–23,23,25,25,28,184^ This pathway exhibits a higher barrier (0.52 eV) than the barrier involved in the isomerization S_1_/ $S_{0}^{\mathrm{iso}}$ MECI. Although less favorable, this pathway provides an additional relaxation channel that regenerates the initially excited *trans* isomer, potentially decreasing the *trans* → *cis* isomerization quantum yield and introducing a dependence on excitation energy and temperature.^20,185,186^

For *cis*-DANS, the pathway 2 → 6 → 10 → 11 involves cyclization (see Figure 3b), a mechanism well documented for stilbene and known to yield the photoproduct 4a,4b-dihydrophenanthrene (DHP),^178,181,182,187–191^ which was also suggested to occur in DANS.^192^ This cyclization pathway exhibits a barrier of 0.22 eV, comparable to that of the isomerization through S_1_/ $S_{0}^{\mathrm{iso}}$ , suggesting that these two relaxation pathways could be competing. At the S_1_/ $S_{0}^{\mathrm{cyc}}$ MECI, the carbon atoms of the two benzene rings adjacent to the double bond move so close together that a new chemical bond can be formed, creating a third six-membered ring (see Figure 3 and third geometry in Figure 1). This close arrangement causes the connected hydrogens to orient out of the molecular plane, consistent with the proposed cyclization mechanism of the parent compound.^178,181,182,187–191^

The two additional novel deactivation routes are discovered by excited-state umbrella sampling and related to cyclization, see Figure 3b. In these two alternative routes, the closing of the rings is accompanied by a pronounced twist of the NO_2_ group, resembling an interaction in which one NO_2_ oxygen is drawn toward the methyl substituents of the dimethylamino moiety. Additionally, these MECIs are characterized by either rotation or pyramidalization of the NO_2_ functional group (S_1_/ $S_{0}^{\mathrm{rot\&cyc}}$ and S_1_/ $S_{0}^{\mathrm{pyr\&cyc}}$ ). Because the TS optimizations for the paths leading to the new NO_2_-involved MECIs (segments 6 → 12 and 6 → 13 in Figure 3b) failed to converge, these TS geometries are not denoted as optimized stationary points in Figure 3b and not the minimal energy pathway but interpolation scan is showed here. The MECI optimizations themselves were successful. Along the minimum-energy pathway for S_1_/ $S_{0}^{\mathrm{rot\&cyc}}$ (segment 2 → 6 → 12) (see in ), there is a region where the S_1_ and S_0_ states approach near-degeneracy. This is also weakly reflected in the interpolation scan in Figure 3b second panel, where the S_0_ state comes closer in energy to S_1_. Optimization initialized from this region relaxes directly to the MECI (geometry 12). The relatively low barrier before this near-degenerate precursor suggests that nonadiabatic relaxation may occur not only at the optimized MECI itself but also along the preceding path segment. This observation underscores the necessity of extensive sampling—even in small molecular systems—to identify not only MECIs and minimum-energy pathways but all energetically relevant routes that govern the relaxation mechanism.

In summary, structure refinements/optimizations are essential for identifying key geometric changes at critical points and for obtaining representative structures of specific CIs and minima. Moreover, potential energy scans help to give an idea which pathways are more relevant. These approaches therefore remain an important part of investigating photorelaxation pathways.

### 5.2 Nonadiabatic Dynamics

The OM2/MRCI dynamics features the internal conversion from the initially excited S_1_ state to the ground state. For convenience, the excited-state populations were fitted by adding S_1_ and S_2_ population together, see Figure 4. We use a two-step kinetic model that distinguishes between an “early” and a “late” S_1_+S_2_ population in order to capture the delayed decay of the S_1_ population without introducing a simple delay offset (which is sufficient in other cases^132,193–195^ ). This sequential model first captures the transition from early to late S_1_+S_2_ states, followed by the decay from the late S_1_+S_2_ to S_0_ (see more details in ).

**Figure 4:**
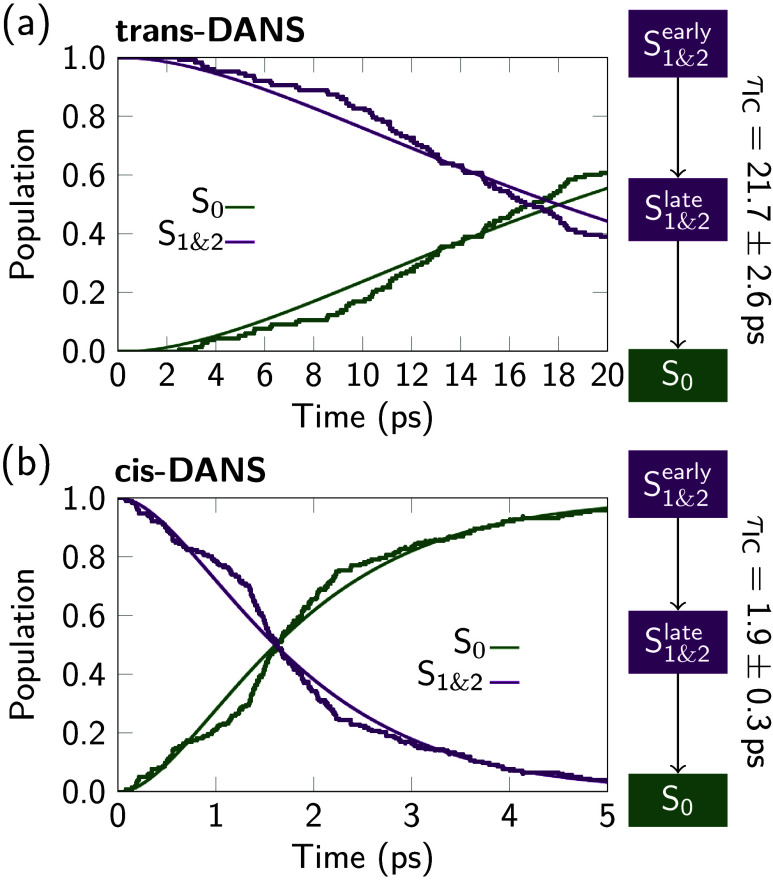
Time-resolved adiabatic populations of DANS in gas phase (thick lines) alongside with fits using sequential first-order kinetic models $A_{0}\overset{\tau_{1}}{\to }A_{1}\overset{\tau_{2}}{\to }A_{2}$ where τ_*IC*_ = τ_1_ + τ_2_ (thin lines) for *trans*-DANS (a) and *cis*-DANS (b). The S_1_ and S_2_ states are grouped together and represented in dark purple, while the ground state is depicted in green. Next to the population panels, the fitted time constants and the corresponding uncertainties (2σ), obtained from bootstrapping,^196^ are shown.

The *trans* isomer (Figure 4a) shows a slower excited-state population decay, consistent with its higher barrier to the CI relative to the *cis*-DANS isomer. A relaxation time constant of 21.7 ± 2.6 ps is obtained, with 61% of the population reaching the ground state after 20 ps. In contrast, the lower excited-state barrier and a Franck–Condon S_1_ energy above the TS separating the excited geometry from the MECIs (Section 5.1) lead to faster relaxation of the *cis* isomer (Figure 4b), allowing simulations to be limited to 5 ps. A slight deviation from the fitted curve in the excited-state population around 1 ps—caused by a brief rise in the S_2_ population—delays decay to the ground state. As a result, the relaxation time constant for *cis*-DANS is 1.9 ± 0.3 ps, with 96% of the trajectories reaching the ground state after 5 ps —approximately an order of magnitude faster than for trajectories starting from the *trans*-isomer.

The mechanism underlying relaxation to the ground state is investigated monitoring several geometrical parameters. The evolution of the central HCCH dihedral angle (Figure 5a-b) serves as a measure of isomerization around the central bond: ϕ_HCCH_ = 0° and ϕ_HCCH_ = 180° correspond to the *cis* and *trans* isomers, respectively. For better visibility, Figure 5 has been extended beyond the total range of 360°.

**Figure 5:**
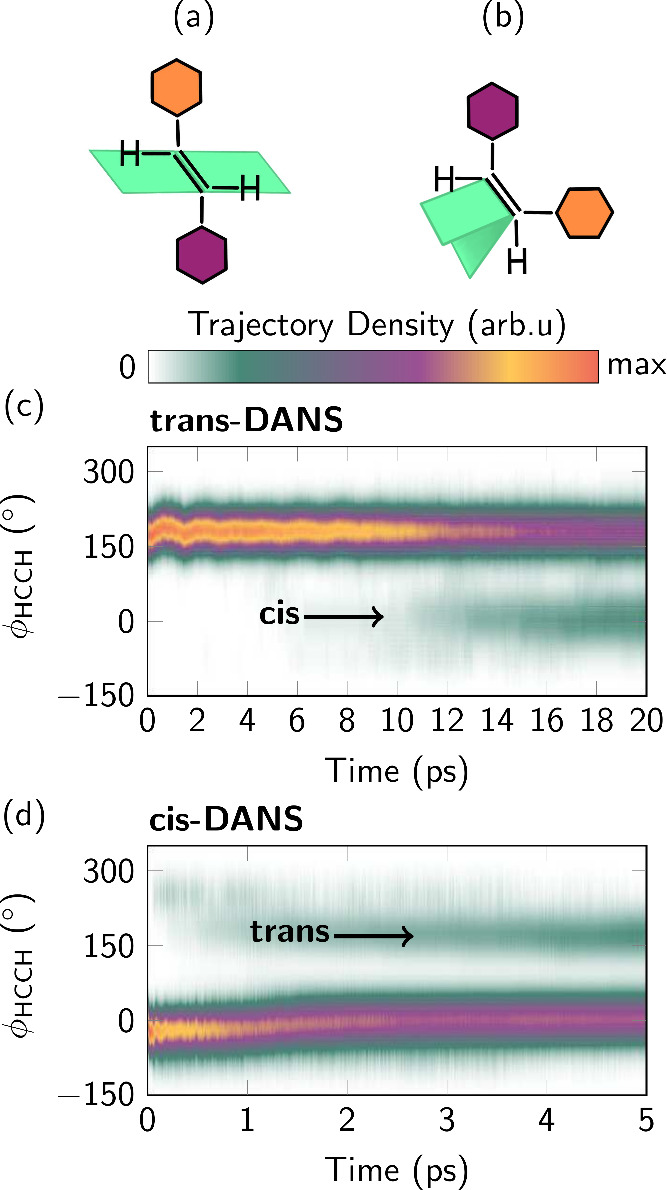
Dihedral angle of the central carbon double bond (HCCH) schematically illustrated for *trans* (a) and *cis* (b) isomers, with the orange hexagon indicating the position of aromatic ring with the NMe_2_, while the purple hexagon represents the aromatic ring with NO_2_ functional group. Time-evolution of the Gaussian-convoluted dihedral angle ϕ_HCCH_ for trajectories starting from the *trans* (c) and *cis* (d) isomers. Black arrows show the low density region, indicating the switching to the other isomer.

The *trans* configuration (Figure 5c) is initially characterized by a HCCH dihedral angle of 150°-160°. A coherent oscillation is observed between 150° and 180°, gradually damping toward 180° around 5 ps. At this point, some trajectories begin to isomerize, as indicated by the emergence of low-density regions (dark green) around 0°. This shift becomes more pronounced after 10 ps, with an increasing number of trajectories falling within the [-20°, 20°] range, signaling the continuous formation of the *cis* isomer. Nevertheless, a substantial fraction of trajectories remains in the *trans* configuration after relaxation to the ground state. During the simulation, 74 trajectories relaxed to the ground state, out of which 31 (42%) isomerized to the *cis* configuration.

In the *cis* isomer (Figure 5d), the HCCH dihedral angle is initially between 0° and −20°. A damped transition from angles slightly below 0° to slightly above 0° can be observed. A low density area (green) emerges already before 1 ps between 150° and 180°, corresponding to the formation of the *trans* isomer. Over the 5 ps simulation, the *trans* population grows gradually. By the end of the simulation, the *cis* configuration still dominates, indicating an isomerization quantum yield lower than 50% (only 44 of 184 trajectories that reached the ground state switched to the *trans* isomer).

We now examine the CIs underlying the dynamics, depicting four geometrical parameters that distinguish the CIs, two associated with each isomer (Figure 6). For the *trans* isomer, we project frames onto planes spanned by the S_1_–S_0_ energy gap and the HCCH dihedral angle (Figure 6a) and the NO_2_ pyramidalization angle (Figure 6b), respectively. The Franck–Condon region highlighted in Figure 6a enables three possible relaxation directions. Two lead to enantiomers of the same CI, differing in the direction of rotation around the central C–C bond and occurring at dihedral angles of approximately 90° and 270° (the latter continuing periodically at −90°). Trajectories hopping at dihedral angles near 90° and 270° pass through the perpendicular-type CI associated with isomerization (S_1_/ $S_{0}^{\mathrm{iso}}$ ). Trajectories hopping around 180° relax through the pyramidalized NO_2_ CI (S_1_/ $S_{0}^{\mathrm{pyr}}$ ). The direction pointing vertically downward from the Franck–Condon region retains the dihedral angle around 180°, corresponding to a relaxation pathway through a different CI, specifically the pyramidalized NO_2_ (S_1_/ $S_{0}^{\mathrm{pyr}}$ ). Additionally, five trajectories immediately hopped to the S_1_ excited state after reaching the ground state, effectively isomerizing to the *cis* isomer in the ground state, and subsequently returned to the ground state via the cyclization CI of the *cis* isomer (S_1_/ $S_{0}^{\mathrm{cyc}}$ ), see trajectories near 0° HCCH dihedral in Figure 6a.

**Figure 6:**
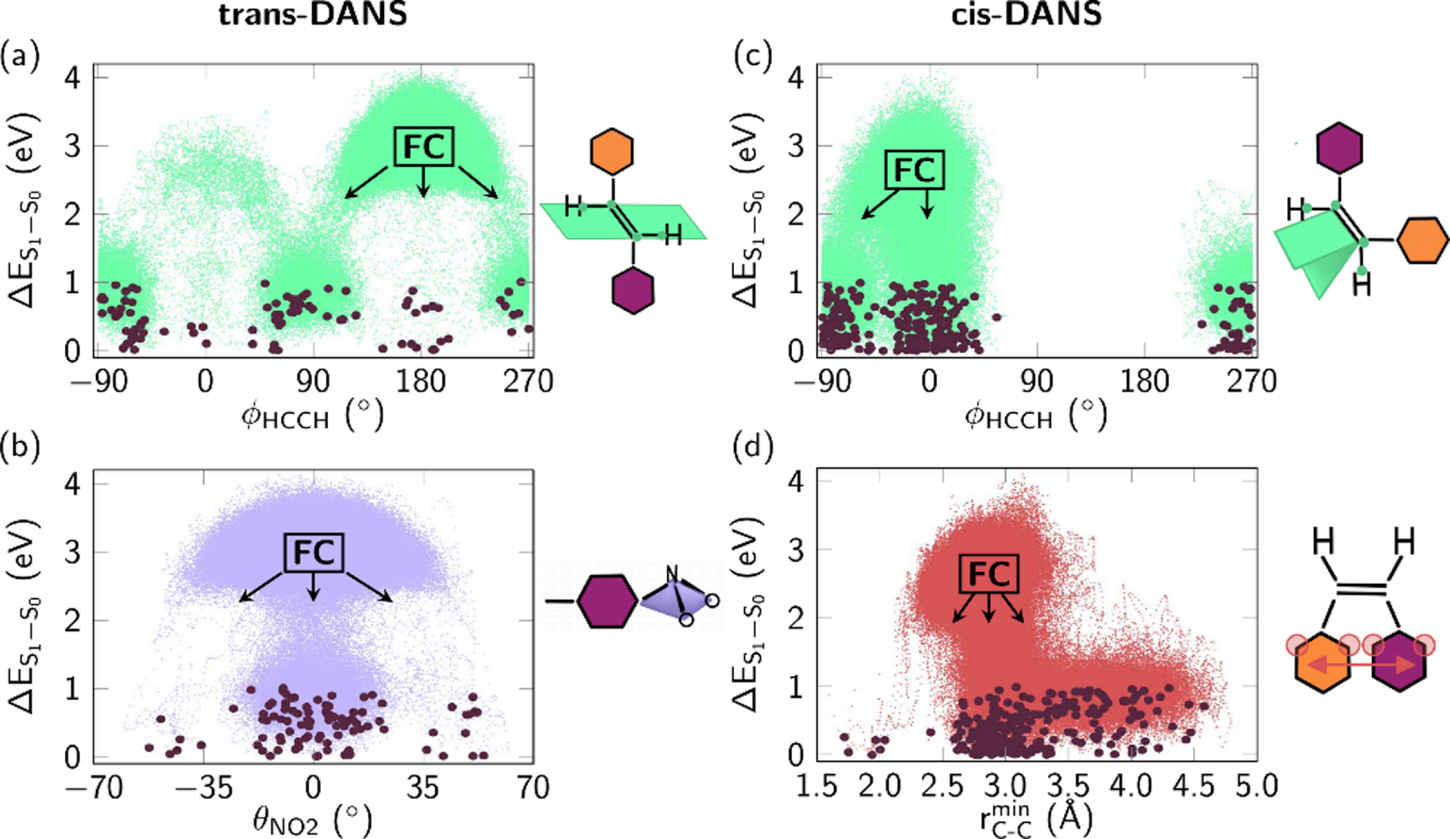
Scatter plot of different internal coordinates versus the S_1_ – S_0_ energy gap. Fine colored points represent frames collected every 1 fs from all trajectories. Larger purple circles indicate geometries at which a hop to ground state occurred. From the Franck–Condon (FC) region, black lines indicate relaxation directions. The colors and drawings to the right of each panel specify the corresponding geometrical parameter: (a) HCCH dihedral angle (green), (b) NO_2_ pyramidalization angle (violet), (c) HCCH dihedral angle (green), and (d) the minimum distance out of four carbon atoms on opposite rings as indicated in the drawing (red).

The three relaxation pathways are also evident when considering NO_2_ pyramidalization (Figure 6b). The perpendicular-type isomerization CIs (S_1_/ $S_{0}^{\mathrm{iso}}$ ) cluster around a pyramidalization angle near zero, while two distinct groups at angles below −35° and above 35° (S_1_/ $S_{0}^{\mathrm{pyr}}$ ) correspond to CIs that return back to the original *trans* isomer via slight twisting and pyramidalization of the NO_2_ moiety. In summary, of the 128 trajectories initialized from the *trans* configuration that conserved the total energy, 78 (61% of 128) reached the ground state within 20 ps: 33 (42% of 78) switched to the *cis*-DANS, while 45 (58% of 78) remained in the *trans* isomer. From the 45, 26 (58%) passed through the S_1_/ $S_{0}^{\mathrm{iso}}$ , and 19 (42%) proceeded via the S_1_/ $S_{0}^{\mathrm{pyr}}$ . Overall, the conversion to the *cis* isomer is less than 50% due to competing relaxation channels back to the *trans* configuration.

For the *cis* isomer, we plot the S_1_–S_0_ energy gap versus the HCCH dihedral angle (Figure 6c) and the minimum distance between four phenyl carbon atoms (two on each ring, Figure 6d). From Figure 6c, two distinct relaxation pathways start from the Franck–Condon region at 0°. One proceeds toward −90° (which continues periodically at 270°), corresponding to the S_1_/ $S_{0}^{\mathrm{iso}}$ CI. Some trajectories that hop at this CI subsequently relax toward the *trans* isomer (toward 180°). Unlike the *trans* isomer, here, rotation around the C–C bond to reach the other isomer is possible in only one direction, as rotation in the opposite direction would bring the rings closer together, possibly leading to a cyclization. Trajectories following the cyclization pathway maintain ϕ_HCCH_ ≈ 0°. Closer inspection of these geometries shows that cyclization predominantly follows the pathway with simultaneous twist of the NO_2_ functional group, through S_1_/ $S_{0}^{\mathrm{rot\&cyc}}$ .

The minimum distance between carbon atoms on the rings, shown in Figure 6d, helps to differentiate the two cyclization routes (cyclization with or without twist of NO_2_). Distances larger than 3.4 Å correspond to isomerization CIs, while distances of 2.5–3.4 Å indicate alternative CIs with NO_2_ twisting, and NO_2_ pyramidalization accompanied by rings getting closer. It is important to note that, when examining these structures with twisted or even pyramidalized NO_2_, we observe a family of slightly different CIs with varying degrees of torsion and ring closeness. This observation underscores that adequate sampling of both the CI region and the pathways leading to it is essential to capture all energetically accessible CIs. A small subset of trajectories shows distances of 1.5–2.0 Å, characteristic of the stilbene-type cyclization through S_1_/ $S_{0}^{\mathrm{cyc}}$ . These short-distance events occur less frequently than the NO_2_-involved CIs.

Of 192 trajectories initialized from the *cis* isomer, 184 (96%) relaxed to the ground state. Of these, 92 (50%) passed through the isomerization CI (S_1_/ $S_{0}^{\mathrm{iso}}$ ), 88 (48%) through the cyclization with NO_2_ twist-type CI (S_1_/ $S_{0}^{\mathrm{NO}2\mathrm{\&cyc}}$ ), and 4 (2%) through the cyclization CI also observed for stilbene (S_1_/ $S_{0}^{\mathrm{cyc}}$ ). The NO_2_-mediated cyclizations are grouped together, as distinguishing between nonoptimized points is not straightforward. Among the trajectories passing through S_1_/ $S_{0}^{\mathrm{iso}}$ , 44 (48%) converted to the *trans* isomer, while 48 (52%) remained in the *cis* form. Of those going through any cyclization CI, exactly half (46 trajectories) displayed tendency to cyclization (keeping short distances of the rings), whereas 46 (50%) did not form a bond and remained in the *cis* configuration.

In conclusion, nonadiabatic dynamics is particularly valuable for obtaining information about nonradiative decay rates, more quantitative insight of the relevant relaxation pathways, and geometric changes along the relaxation process. In addition, it can serve as an exploratory tool to identify new CIs that can subsequently be used as starting points for further optimization.

### 5.3 Umbrella Sampling in the First Excited State

Up to this point, we have obtained relaxation time constants, tracked dihedral evolution during isomerization, and identified alternative relaxation pathways for both isomers (Figures 4–6). Analysis of trajectory distributions provides a qualitative sense of relative energy barriers, but the nonadiabatic dynamics alone cannot yield quantitative barrier heights, motivating the use of excited-state umbrella sampling. Furthermore, barriers obtained from static scans, such as those shown in Figure 3 do not fully capture the complexity of the critical points, where multiple important conformations may exist. To overcome this structural complexity, we turned to adiabatic excited-state enhanced sampling using the energy gap between S_1_ and S_0_ as the CV, and thus obtain free-energy, internal-energy, and entropy profiles.

The thermodynamic profiles are used to further explain the nonadiabatic dynamics observed in Section 5.2. Since we perform adiabatic simulations in the S_1_ state, the profiles do not directly reflect the nonequilibrium photoprocess induced by photoexcitation. However, they allow for a more detailed characterization of the S_1_ PES and to identify which barriers the excited-state trajectories encounter, and why certain reaction paths are preferred over others, which is information that may not be accessible from the minimum energy scans presented in Section 5.1. We note that this interpretation is meaningful as long as barriers with Δ*F* ^‡^≫ *k*_B_ *T* are present, so that the molecule spends sufficient time in the excited state to form a pseudoequilibrium. Additionally, even in a nonequilibrium process, the system must traverse the same regions of configuration space, and the entropic decomposition reveals features that are invisible to minimum energy path methods. Barrierless processes that proceed on ultrafast time scales do not benefit from such an analysis. A further practical criterion is time scale separation: if the nonadiabatic relaxation is slow compared to the local equilibration time within each umbrella window, which can be assessed by comparing the autocorrelation time of the CV to the overall relaxation time scale from nonadiabatic molecular dynamics, the quasi-equilibrium assumption is justified. In the present case, the decorrelation time in the umbrella windows are tens of femtoseconds or even less (see in ), whereas the relaxation time scales shown in Figure 4 are in the picosecond regime, and therefore well separated by several orders of magnitude.

#### 5.3.1 Thermodynamic Profiles

Figure 7a-c shows the thermodynamic profiles for the *trans* isomer as a function of the energy gap: large energy gaps correspond to geometries near the S_1_ minimum and as the gap decreases, the system is approaching a CI. Sampling was extended up to 3.3 eV to confirm that the S_1_ minimum occurs at an energy gap of 3.0 eV, as evidenced by the continued rise of the free-energy profile beyond this value. In Figure 7a, the free-energy difference between the minimum at 3 eV energy gap and local maximum at 2.3 eV is about 8.2 kcal/mol (0.36 eV). This quantity should not be interpreted as an activation free energy, which would require the integration over the full marginal Boltzmann distribution.^197^ This barrier from the sampling closely matches the barrier height (0.33 eV) determined from static calculations in Figure 3. After crossing this barrier, a second, broad minimum appears at an energy gap of 1 eV, where the system can become temporarily trapped before reaching the CI. Similar low-energy regions are observed in the potential energy scans preceding the MECIs in Figure 3 (see region before critical points 5 and 9).

**Figure 7:**
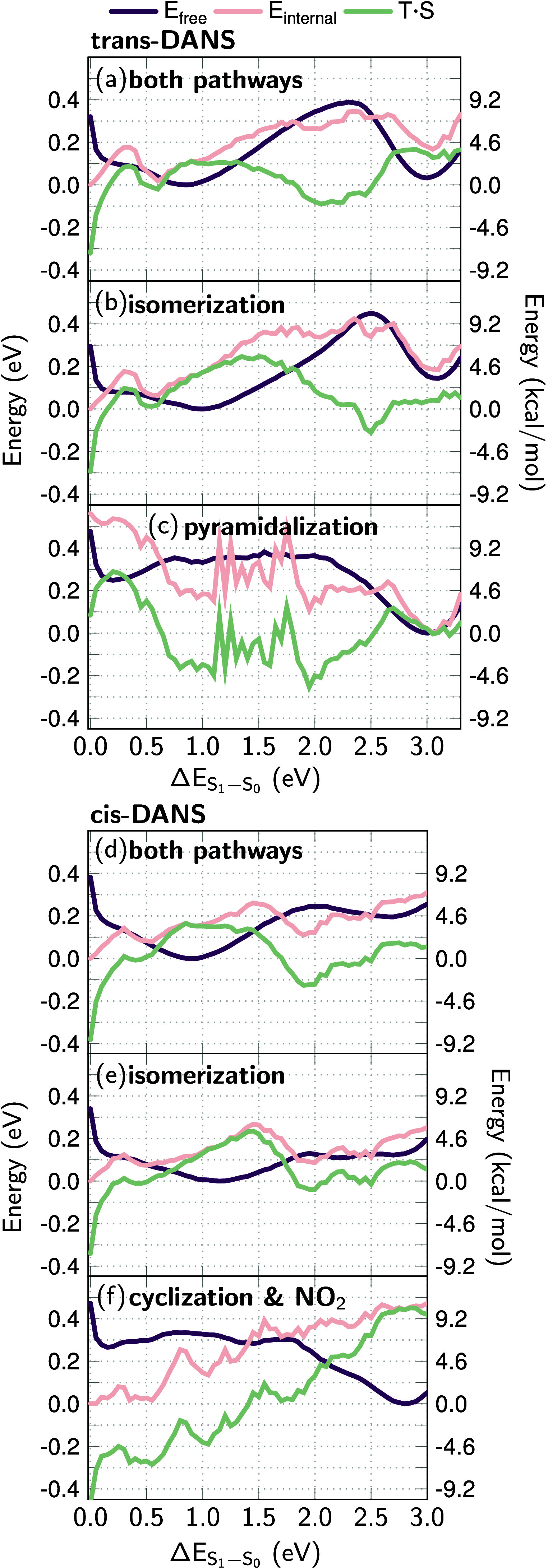
Free energy (E_free_, purple), internal energy (E_internal_, pink), and entropy (T·S, light-green) for *trans* ((a), (b), and (c)) and *cis* ((d), (e), and (f)) isomers, as obtained from excited-state umbrella sampling with $\Delta E_{S_{1}-S_{0}}$ as a collective coordinate.

Figure 7b excludes data corresponding to the pyramidalization pathway to focus on the isomerization pathway that proceeds via the S_1_/ $S_{0}^{\mathrm{iso}}$ CI. Since the free-energy barrier observed around 2.3 eV is comparable to that in the combined pathway (Figure 7a), the ∼ 8 kcal/mol barrier can be attributed to isomerization. Differences in barrier width in Figures 7a and b imply that isomerization and pyramidalization occur at different energy gaps, resulting in a broader barrier when both pathways are combined.

Finally, Figure 7c, excludes isomerization to focus on the pyramidalization path. Unfortunately, the umbrella sampling simulations (which have a constant number of samples in each window for both pathways) did not accurately sample the latter pathway in the region between 1.10 and 2.0 eV, introducing large oscillations in the entropy and internal energy, which prevents defining a barrier in that interval. The roughness of the free-energy profile in this region indicates insufficient sampling, which causes the harder to converge profiles (entropy and internal energy) to fluctuate wildly. Nevertheless, separating the two pathways still reveals qualitatively significantly different behavior. Interesting is though that the free-energy profile differs from that of the isomerization, showing a continuous increase with no second minimum prior to the CI region.

In Figures 7a and b, the entropy decreases significantly at $\Delta E_{S_{1}-S_{0}}\approx 2.3$ eV, which can be explained by the reduction in accessible molecular configurations. Approaching the barrier, the molecular system becomes confined into a specific orientation required to surpass the energy barrier, thereby limiting accessible vibrational and rotational modes, which reduces the entropy. Left from the barrier, near an energy gap of approximately 1.0 eV, the entropy increases again as the free-energy profile reaches a second minimum and the internal energy decreases. Interestingly, for $\Delta E_{S_{1}-S_{0}}$ decreasing below 1.0 eV, both entropy and internal energy profile exhibit a local minimum followed by a local maximum, the effects canceling each other out and leading only to a slight increase in the free energy profile toward smaller gap values.

For *cis*-DANS, the free energy profile of Figure 7d exhibits a shallow minimum for large $\Delta E_{S_{1}-S_{0}}$ , which is separated by only a small barrier (difference between the local extrema) of approximately 1 kcal/mol (0.04 eV) from configurations with a smaller energy gap. By separating the two relaxation pathways of the *cis* isomer—isomerization (panel e) and cyclization (panel f)—clear differences in the free-energy profiles emerge. In the isomerization, the shallow high energy gap minimum nearly vanishes, resulting in an almost flat profile for energy gaps above 2.0 eV and a broad local minimum centered around 1.2 eV. In contrast, when considering all cyclization events together (predominantly the NO_2_-twist variant), the opposite trend is observed: a pronounced minimum for large $\Delta E_{S_{1}-S_{0}}$ values with an increase in free energy by about 8 kcal/mol (0.35 eV) toward smaller gaps and then an almost flat profile up to close to the CI. As mentioned in Section 5.2, we have identified not only the cyclization pathway observed for the parent molecule but additional ones involving a cyclization with twisting NO_2_ and with the NO_2_ pyramidalization. This diversity of pathways demonstrates that umbrella sampling is required to capture the full ensemble of CI variants. The corresponding energy profiles illustrate this ensemble, including CI configurations that do not lead to isomerization.

The unbiased dynamics simulations confirm the presence of the minimum around 1.0 eV found in the free-energy profiles for isomerization pathway for both isomers (). In contrast to the isomerization, the cyclization path displays a continuous entropy decrease with continuous decrease of the energy gap. This comparison highlights the strength of the free-energy analysis: while the PES scans in Figure 3 suggest comparable barriers for both pathways, the free-energy profiles show a distinct behavior for the cyclization pathway, where ring closure restricts molecular motion, leading to a decrease in entropy and increase in free energy.

Interestingly, as the system approaches the CI, the free energy rises sharply accompanied by a corresponding drastic decrease in entropy (Figure 7). This phenomenon originates from the reduced effective dimensionality near, where the system is confined to 3*N* – 8 degrees of freedom, for *N* atoms. The degeneracy conditions imposed by the branching-plane vectors (*g* and *h*) constrain the nuclear geometry, severely restricting the accessible configurational space and thereby lowering the entropy. If the energy gap can be parametrized exclusively by the branching vectors,^198^ independent of all other degrees of freedom, then it can be shown that the entropy and therefore also the free-energy profile diverge (as ∝ ln­(Δ*E*), see ref 76 for a detailed derivation). That the free energy tends to infinity is significant, as this means that transitions between electronic states will occur at small but nonzero energy gaps in simulations where nuclei are treated classically,^75^ () as the precise CI is inaccessible, which makes the shape of the free-energy landscape especially valuable for interpreting surface hopping behavior.

#### 5.3.2 Distribution of Configurations

In Figure 8a-b the frames from the umbrella sampling simulations performed in the S_1_ state are projected onto the central dihedral angle (ϕ_HCCH_) and energy gap $\left(\Delta E_{S_{1}-S_{0}}\right)$ . The sampling data was also projected onto additional geometric parameters see in , including the NO_2_ pyramidalization and distances between carbon atoms of the rings. For both isomers, at least two distinct CIs can be identified in Figure 8a-b, as previously shown in Figure 3; one set of geometries maintains the dihedral angle of the parent compound (corresponding to the pyramidalization and cyclization CIs for *cis* and *trans*, respectively), while a different set can be found for configurations corresponding to the isomerization CI.

**Figure 8:**
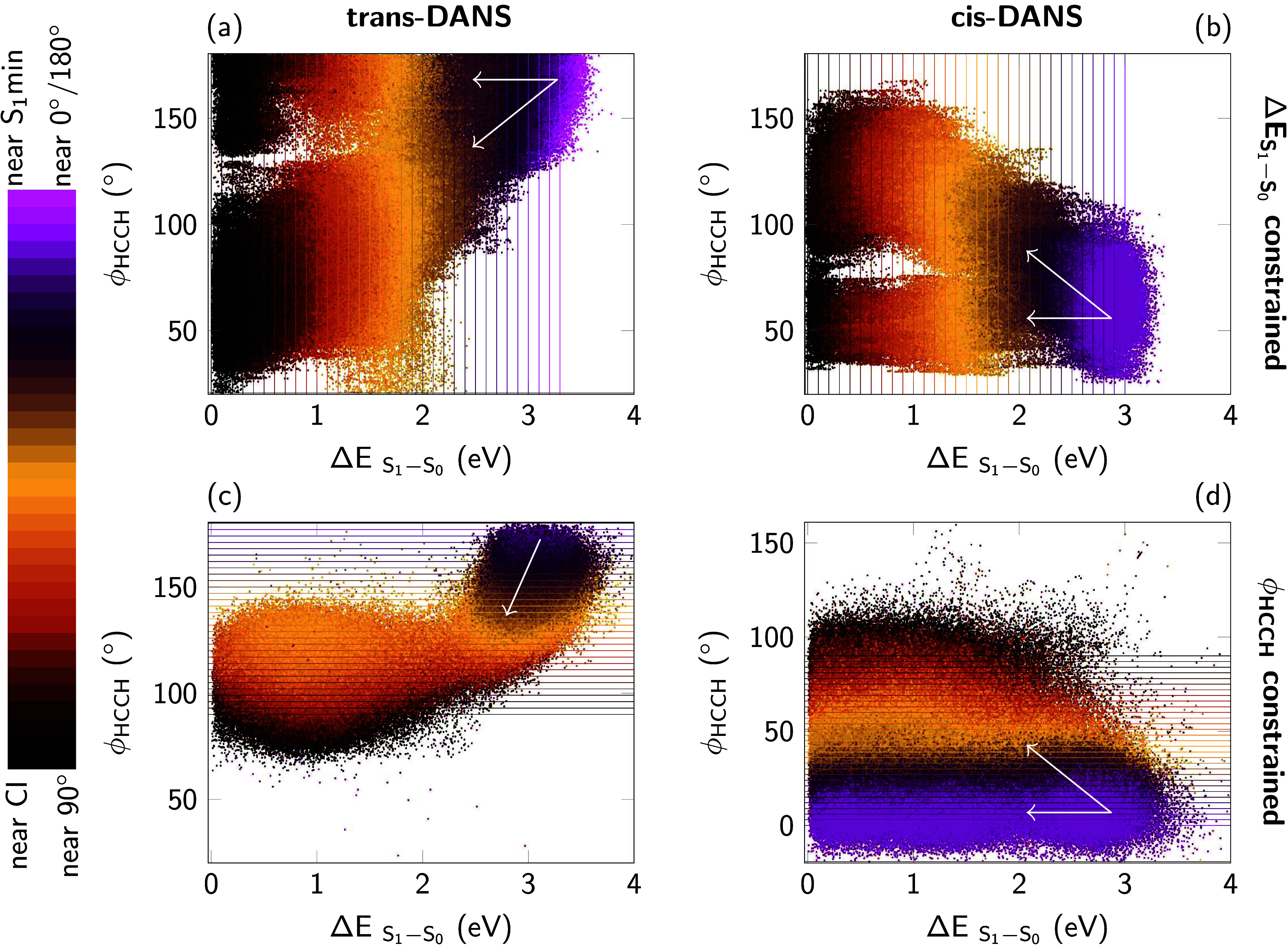
Projection of the simulation frames onto the central dihedral angle ϕ_HCCH_ and the S_1_/S_0_ energy gap for both isomers. (a)/(b) results obtained using the energy gap $\Delta E_{S_{1}-S_{0}}$ as CV. (c)/(d) results from simulations with ϕ_HCCH_ as CV. Vertical and horizontal lines indicate the centers of the applied bias potentials. White arrows indicate the starting point of the sampling and the directions toward the CIs.

For *trans*-DANS (Figure 8a), one CI occurs near 180°, the S_1_/ $S_{0}^{\mathrm{pyr}}$ and the other at 90°, the S_1_/ $S_{0}^{\mathrm{iso}}$ . The pathway toward the S_1_/ $S_{0}^{\mathrm{iso}}$ CI is sampled more extensively, likely because the barrier for pyramidalization of the NO_2_ group is significantly higher according to static potential energy scans (0.53 eV vs 0.33 eV). In the case of the *cis* isomer (Figure 8b), both CIs, S_1_/ $S_{0}^{\mathrm{iso}}$ and S_1_/ $S_{0}^{\mathrm{cyc}}$ , and their associated pathways are clearly visible and well-sampled. The two pathways are clearly distinguishable: one maintains the initial HCCH dihedral angle around 0°, while the other progresses toward 90° and beyond.

For comparison, umbrella sampling was also performed using the HCCH dihedral angle as CV for both isomers and the obtained frames are also projected onto the dihedral angle and the S_1_/S_0_ energy gap, as shown in Figure 8c-d. For completeness, we plot these results also in the space of S_1_/S_0_ energy gap and the central CCCC diehedral angle in . These figures illustrate why the energy gap serves as a more effective CV than the dihedral angle for exploring the CI region and associated relaxation pathways. When constraining the dihedral angle, the introduced bias restricts the sampled conformations primarily to rotations around the bridging CC bond. As demonstrated in Figure 8c for the *trans* isomer, this approach samples only configurations with a small energy gap near the isomerization CI region, while never visiting the pyramidalization CI. Constraining the angle at approximately 90° appears to be insufficient to focus the sampling on the CI, as configurations with this dihedral angle have energy gaps of up to 2 eV. In principle, constraining an internal degree of freedom may not lead to a CI at all, especially if the S_1_ minimum lies lower in energy along other degrees of freedom than the one that is fixed. In contrast, using the energy gap as the CV provides more precise control, effectively guiding the sampling toward an actual intersection region.

For the *cis* isomer (Figure 8d), both pathways (isomerization and cyclizations) are sampled due to the proximity of the starting geometry to the cyclic *cis* structure, as can be observed that the purple color at 0° ranges over entire energy gap from approximately 3 to 0 eV. Similar to the *trans* isomer, the dihedral angle windows near 90° (black color) exhibit a large spread for the energy gaps (0 to approximately 2 eV). Thus, the sampling includes structures that are not necessarily near the CI, and as a result, the free-energy profiles constructed using dihedral angle constraints do not accurately represent the reaction pathways from the S_1_ minima toward the CI (). However, when these results are projected onto the S_1_/S_0_ energy gap, the characteristic free-energy increase is recovered ().

This section demonstrated the advantages of using the energy gap as a CV for excited-state umbrella sampling. By explicitly capturing entropy contributions, characteristic features in the free-energy profiles—such as increases at TSs or CIs as well as during cyclization—could be explained. Multiple relevant CIs for each isomer and their associated free-energy pathways were identified, highlighting why the energy gap is more informative than dihedral angles as a CV for exploring excited-state relaxation pathways. Moreover, this free-energy framework provides insight into the most probable geometries where transitions occur.

## 6 Conclusions

In this work, we presented a workflow for studying photorelaxation processes, using the photoswitch 4-(N,N-dimethylamino)-4′-nitrostilbene (DANS) as an example. We applied the OM2/MRCI method for the underlying electronic structure calculations. First, we calculated static potential energy scans, followed by nonadiabatic dynamics simulations using surface hopping and finally, the relaxation pathway from the S_1_ minimum to various CIs was explored through the newly implemented excited-state umbrella sampling. The latter provided insight into relaxation pathways in cases where static potential energy surface scans were insufficient and proper sampling of the relaxation path was essential.

The full protocol applied to DANS in gas phase delivered five CIs: An isomerization CI, accessible from both the *trans* and *cis* isomers; an NO_2_-pyramidalization CI for the *trans* isomer; and three cyclization CIs, two with and one without NO_2_ twist for the *cis* isomer. For the *trans* isomer, we confirmed the presence of a CI associated with vibrational modes of the NO_2_ group, as suggested by experimental studies,^22,23,25^ although the barrier calculated from the potential energy surface for this pathway is higher than that of the isomerization route. For the *cis* isomer, a cyclization pathway consistent with findings for the parent compound stilbene,^178,181,182,187–191^ and two new (to our knowledge previously unreported) cyclization pathway accompanied by NO_2_ twist was shown to be a competing relaxation channel. We found that the *trans* isomer relaxes significantly more slowly than the *cis* isomer (21.7 ps vs 1.9 ps), which can be attributed to a smaller energy barrier that separates the S_1_ minimum from the CIs in the *cis* case.

Excited-state umbrella sampling using the energy gap as a CV, combined with the free-energy and entropy formalism described in ref 118, provided detailed insight into the properties along the relaxation pathway. The calculated free-energy profiles also revealed distinctive features of the isomerization pathway, highlighting how it differs from the other relaxation pathways and providing insight into the corresponding entropy profile. For both isomers in the isomerization path, a second minimum appears in the S_1_ state after crossing the barrier. Another notable feature is the sharp increase in free energy accompanied by a decrease in entropy as the system approaches the CI, presenting that the transition between electronic states in surface hopping will occur at small but nonzero energy gaps.^76^

This work demonstrated how excited-state umbrella sampling for exploring relaxation pathways can be useful, particularly when using the energy gap as the CV, instead of a geometric parameter. The energy gap provides a natural and direct means of following the relaxation process, allowing targeted sampling along the reaction pathway rather than across the entire configuration space. Further, it offers control over sampling in both large and small energy gap regions. Umbrella sampling may be especially suited for nonadiabatic enhanced sampling,^112^ as methods like metadynamics apply state-specific bias potentials, making them less appropriate when the system evolves across multiple electronic states.

For small gas-phase molecules with few degrees of freedom, static potential energy calculations often provide a reasonable approximation to free-energy profiles, as observed in DANS. In contrast, larger systems—particularly in the presence of environmental effects—exhibit a much more intricate relaxation landscape, with numerous excited-state minima, barriers, and MECIs. In these cases, sampling-based approaches such as umbrella sampling become essential to properly account for entropic contributions and to identify the most relevant relaxation pathways.

In analogy to its established role in ground-state sampling studies of solvated and biological systems,^199–203^ extending umbrella sampling to excited-state processes offers a powerful strategy to elucidate photochemical mechanisms in complex environments, where environmental interactions can redirect pathways toward CIs and ultimately determine relaxation outcomes. In this context, the approach presented here is particularly well suited for implementation within a QM/MM framework, enabling an explicit description of environmental effects. Building on previous studies of DANS on amorphous silica surfaces,^164,204^ future work will be devoted to investigate dynamics in a glass surface.

## Supplementary Material





## Notes

Supporting Information contains (i) background on the implemented collective variables in SHARC, (ii) a step-by-step guide to umbrella sampling, (iii) a discussion of the excitation space and computed absorption spectrum, (iv further details on the scans along the relaxation pathways in the excited state and the fitting of the electronic populations, (v) further results on decorrelation times, (vi) comparison of PMF and free energy profiles, (vii) distribution of energy gaps at hops during the nonadiabatic dynamics simulations.(PDF)
The compressed file (.zip) contains template files and example trajectories for the nonadiabatic dynamics and excited-state umbrella sampling.(ZIP)
