# Supplementary material for: Umbrella Sampling for Excited States Using a Semiempirical Method
Source: JACS Au. 2026 May 18;6(6):3547–69. doi: 10.1021/jacsau.6c00302 (PMC13291962; doi:10.1021/jacsau.6c00302)
Supplement: Supplementary file 1 [file au6c00302_si_001.pdf]

Supporting Information: Umbrella Sampling for Excited States using a Semiempirical Method

Dóra Vörös,[†,¶] Hans Georg Gallmetzer,[†,‡] Johannes C. B. Dietschreit,[†] Sebastian Mai,[*,†] and Leticia González[*,†]

†*Institute of Theoretical Chemistry, Faculty of Chemistry, University of Vienna, Währinger Straße 17, 1090 Vienna, Austria.*
‡*Doctoral School in Chemistry(DoSChem), University of Vienna, 1090 Vienna, Austria*
¶*Vienna Doctoral School in Physics, University of Vienna, Boltzmanngasse 5, 1090 Vienna, Austria.*

E-mail: sebastian.mai@univie.ac.at; leticia.gonzalez@univie.ac.at

Contents

S1 Implemented CVs and their Gradients S-2

S2 Step-by-step Guide for Umbrella Sampling S-4

S3 Excitations Space explained S-6

S4 Static Scan of Excited-State Relaxation Pathways S-9

S5 Excited-state population fitting S-11

S6 Decorrelation Timescales S-13

S7 Nonadiabatic dynamics and excited-state umbrella sampling S-15

S8 Energy Gap Distribution At Hop S-17

S1 Implemented CVs and their Gradients

Here we summarize the equations that are applied to calculate the energy biases and corresponding gradients for distance, angle, and dihedral angle constraints in umbrella sampling simulations.

Distance used as constraint:

Distance between two points, defined as the Euclidean norm of their vector difference::

$$R_{ij} = ||\mathbf{R}_i - \mathbf{R}_j||_2 \tag{1}$$

The following bias potential is introduced:

$$E_{\text{bias}} = \frac{k}{2}(R_{ij} - R_{\text{target}})^2. \tag{2}$$

Corresponding gradient with respect to atom i:

$$\nabla_{\mathbf{R}_i} E_{\text{bias}} = k\frac{(R_{ij} - R_{\text{target}})}{R_{ij}}(\mathbf{R}_i - \mathbf{R}_j) \tag{3}$$

Corresponding gradient with respect to atom j:

$$\nabla_{\mathbf{R}_j} E_{\text{bias}} = -k\frac{(R_{ij} - R_{\text{target}})}{R_{ij}}(\mathbf{R}_i - \mathbf{R}_j) \tag{4}$$

Angle used as constraint:

To define vectors and angles we have the following points:

$$\mathbf{R}_i, \quad \mathbf{R}_j, \quad \mathbf{R}_k, \tag{5}$$

they define two bond vectors

$$\mathbf{R}_1 = \mathbf{R}_i - \mathbf{R}_j, \qquad \mathbf{R}_2 = \mathbf{R}_k - \mathbf{R}_j, \tag{6}$$

and they form the following angle θ

$$\theta = \arccos\left(\frac{\mathbf{R}_1 \cdot \mathbf{R}_2}{||\mathbf{R}_1||||\mathbf{R}_2||}\right). \tag{7}$$

The following bias potential is introduced:

$$E_{\text{bias}} = \frac{k}{2}(\theta - \theta_{\text{target}})^2. \tag{8}$$

Derivative of the cosine of the angle for atom i:

$$\frac{\partial \cos\theta}{\partial \mathbf{R}_i} = \frac{1}{||\mathbf{R}_1||}\left(\frac{\mathbf{R}_2}{||\mathbf{R}_2||} - \frac{\mathbf{R}_1}{||\mathbf{R}_1||}\cos\theta\right) \tag{9}$$

Derivative of the cosine of the angle for atom k:

$$\frac{\partial \cos\theta}{\partial \mathbf{R}_k} = \frac{1}{||\mathbf{R}_2||}\left(\frac{\mathbf{R}_1}{||\mathbf{R}_1||} - \frac{\mathbf{R}_2}{||\mathbf{R}_2||}\cos\theta\right) \tag{10}$$

Corresponding gradient with respect to atom i:

$$\nabla_{\mathbf{R}_i} E_{\text{bias}} = -\frac{k(\theta - \theta_{\text{target}})}{\sin\theta} \frac{\partial \cos\theta}{\partial \mathbf{R}_i} \tag{11}$$

Corresponding gradient with respect to atom k:

$$\nabla_{\mathbf{R}_k} E_{\text{bias}} = -\frac{k(\theta - \theta_{\text{target}})}{\sin\theta} \frac{\partial \cos\theta}{\partial \mathbf{R}_k} \tag{12}$$

Corresponding gradient with respect to atom j:

$$\nabla_{\mathbf{R}_j} E_{\text{bias}} = -(\nabla_{\mathbf{R}_i} E_{\text{bias}} + \nabla_{\mathbf{R}_k} E_{\text{bias}}) \tag{13}$$

Dihedral angles used as constraint:
To define vectors and angles we have the following points:

$$\mathbf{R}_i, \quad \mathbf{R}_j, \quad \mathbf{R}_k, \quad \mathbf{R}_l, \tag{14}$$

they define three bond vectors

$$\mathbf{R}_1 = \mathbf{R}_i - \mathbf{R}_j, \qquad \mathbf{R}_2 = \mathbf{R}_k - \mathbf{R}_j, \qquad \mathbf{R}_3 = \mathbf{R}_l - \mathbf{R}_k, \tag{15}$$

Next, form two normalized normal vectors:

$$\mathbf{m} = \frac{\mathbf{R}_1 \times \mathbf{R}_2}{||\mathbf{R}_1 \times \mathbf{R}_2||} \tag{16}$$

$$\mathbf{n} = \frac{\mathbf{R}_2 \times \mathbf{R}_3}{||\mathbf{R}_2 \times \mathbf{R}_3||} \tag{17}$$

The dihedral (torsion) angle ϕ is then defined as:

$$\phi = \arccos \mathbf{m} \cdot \mathbf{n} \tag{18}$$

The following bias potential is introduced:

$$E_{\text{bias}} = \frac{k}{2}(\phi - \phi_{\text{target}})^2. \tag{19}$$

The derivative of the energy with respect to each atomic coordinate in general ($\alpha \in i, j, k, l$) looks like:

$$\nabla_{\mathbf{R}_\alpha} E = \frac{\partial E_{\text{bias}}}{\partial \phi} \frac{\partial \phi}{\partial \mathbf{R}_\alpha}, \tag{20}$$

with

$$\frac{\partial E_{\text{bias}}}{\partial \phi} = k(\phi - \phi_{\text{target}}), \tag{21}$$

and because the dihedral is computed via an arccosine function:

$$\frac{\partial \phi}{\partial \mathbf{R}_\alpha} = -\frac{1}{\sin\phi} \frac{\partial (\cos\phi)}{\partial \mathbf{R}_\alpha} \tag{22}$$

Thus, the gradients for each atom are written as:

$$\nabla_{\mathbf{R}_i} E = -\frac{k(\phi - \phi_{\text{target}})}{\sin\phi} \frac{\partial(\cos\phi)}{\partial \mathbf{R}_i}, \tag{23}$$

$$\nabla_{\mathbf{R}_j} E = -\frac{k(\phi - \phi_{\text{target}})}{\sin\phi} \frac{\partial(\cos\phi)}{\partial \mathbf{R}_j}, \tag{24}$$

$$\nabla_{\mathbf{R}_k} E = -\frac{k(\phi - \phi_{\text{target}})}{\sin\phi} \frac{\partial(\cos\phi)}{\partial \mathbf{R}_k}, \tag{25}$$

$$\nabla_{\mathbf{R}_l} E = -\frac{k(\phi - \phi_{\text{target}})}{\sin\phi} \frac{\partial(\cos\phi)}{\partial \mathbf{R}_l}. \tag{26}$$

with

$$\frac{\partial(\cos\phi)}{\partial \mathbf{R}_i} = \frac{(((\mathbf{m} \times \mathbf{n}) \times \mathbf{m}) \times \mathbf{R}_2)}{\|\mathbf{R}_1 \times \mathbf{R}_2\|} \tag{27}$$

$$\frac{\partial(\cos\phi)}{\partial \mathbf{R}_j} = \frac{(((\mathbf{n} \times \mathbf{m}) \times \mathbf{n}) \times \mathbf{R}_3)}{\|\mathbf{R}_2 \times \mathbf{R}_3\|} - \frac{(((\mathbf{m} \times \mathbf{n}) \times \mathbf{m}) \times (\mathbf{R}_1 + \mathbf{R}_2))}{\|\mathbf{R}_1 \times \mathbf{R}_2\|} \tag{28}$$

$$\frac{\partial(\cos\phi)}{\partial \mathbf{R}_k} = \frac{(((\mathbf{m} \times \mathbf{n}) \times \mathbf{m}) \times \mathbf{R}_1)}{\|\mathbf{R}_1 \times \mathbf{R}_2\|} - \frac{(((\mathbf{n} \times \mathbf{m}) \times \mathbf{n}) \times (\mathbf{R}_3 + \mathbf{R}_2))}{\|\mathbf{R}_2 \times \mathbf{R}_3\|} \tag{29}$$

$$\frac{\partial(\cos\phi)}{\partial \mathbf{R}_l} = \frac{(((\mathbf{n} \times \mathbf{m}) \times \mathbf{n}) \times \mathbf{R}_2)}{\|\mathbf{R}_2 \times \mathbf{R}_3\|} \tag{30}$$

S2 Step-by-step Guide for Umbrella Sampling

A practical umbrella sampling workflow consists of the following steps:

1. A CV must be chosen. For excited-state umbrella sampling, we recommend an energy gap as CV, which can in principle be defined between any electronic states, provided that the underlying electronic structure method remains reliable in the relevant region. If the goal is instead to sample a specific geometric change, a geometric coordinate can also be used as CV.

2. The sampling region must be defined, that is, the range of the chosen coordinate and the pathway to be covered. If the initial sampling does not cover the full region of interest, additional calculations can be added later.

3. Starting geometries for the umbrella windows must be generated. If nonadiabatic dynamics simulations are already available, suitable initial structures can be selected from regions where the energy gap matches the desired window values. If such trajectories are not available, the required structures can be generated by sampling from different initial geometries toward the target energy-gap values or by running a set of exploratory trajectories and selecting appropriate structures from them.

4. Short trial simulations with different force constants are recommended in order to estimate the width of the sampled distributions and to adjust the biasing strength accordingly (for starting value we recommend $k = \frac{k_B T}{\sigma^2}$ where σ is the targeted standard deviation). For steep potential energy surfaces, where manual parameter selection may become difficult, automated refinement schemes such as OGRe[174] may provide a useful strategy.

5. Before running the full set of umbrella sampling trajectories, it is important to decide which program will be used to reconstruct the energy profiles, since this determines which quantities need to be stored. The ASE interface can then be set accordingly to write the required data (e.g. energy and gradient of the selected states) for the chosen reconstruction scheme.

6. The umbrella sampling trajectories are run for all windows.

7. The trajectories should be checked for crashes or insufficient sampling. If necessary, additional trajectories should be run such that the amount of usable data is approximately balanced across the windows.

8. The required data are collected for energy reconstruction, noting that the exact quantities needed depend on the reconstruction program employed.

9. The energy profiles are reconstructed from the collected data.

S3 Excitations Space explained

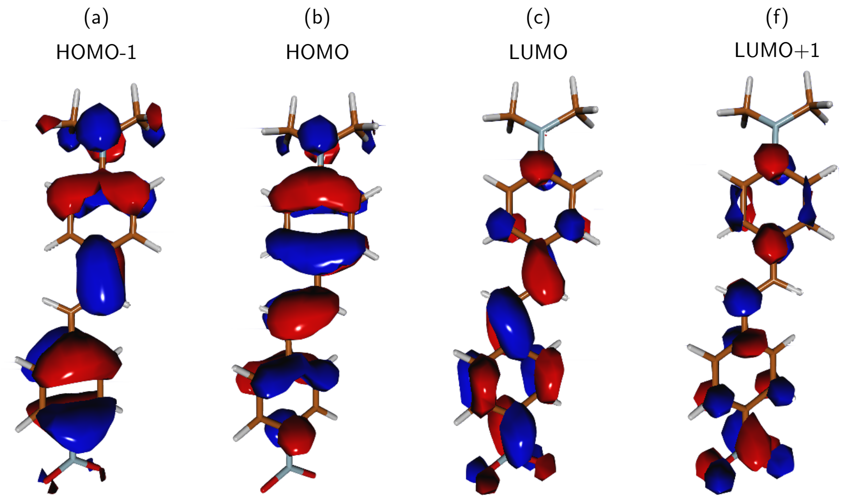

Figure S1: Orbitals included in the excitation space at OM2 level of theory.

The orbitals that were included in the excitation space for the OM2 electronic structure calculations are shown in Figure S1. The HOMO and LUMO orbitals are responsible for the S_1 excitation and give rise to the pronounced charge-transfer character of this state. Based on previous studies,[164] the S_2 state is expected to be a dark $n\pi^*$ state. In our framework, including the orbitals shown in Figure S1 was enough to accurately describe the S_1 state, which is the relevant excited state for the photoexcitation of DANS. However, during the nonadiabatic dynamics with OM2/MRCI, it became evident that not only the character but also the energetic separation of the orbitals must be considered. The four selected orbitals were well separated in energy from the remaining orbitals. When further orbitals were added, due to orbital reordering, other orbitals could enter the excitation space. This resulted in instabilities that caused the dynamics simulations to fail and therefore required the exclusion of the lone-pair orbitals.

In Figure S1, the HOMO-1 is also a π orbital, delocalized over the entire conjugated system, while the LUMO+1 is more localized on the NO_2 group. These orbitals contribute to the second excited singlet state, S_2. We chose to continue with the excitation space including the four orbitals presented in Figure S1 because these are well-separated in energy for DANS.

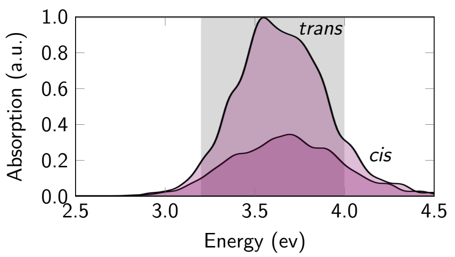

Figure S2: Computed absorption spectrum of *trans*- and *cis*-DANS based on 1000 initial conditions sampled from a Wigner distribution. The gray box highlights the excitation window (3.2–4.0 eV) within the most intense S_1 absorption band. The spectrum is normalized to the maximum intensity of the spectrum of the *trans* isomer. Further information about absorption spectrum of DANS see in Ref 164.

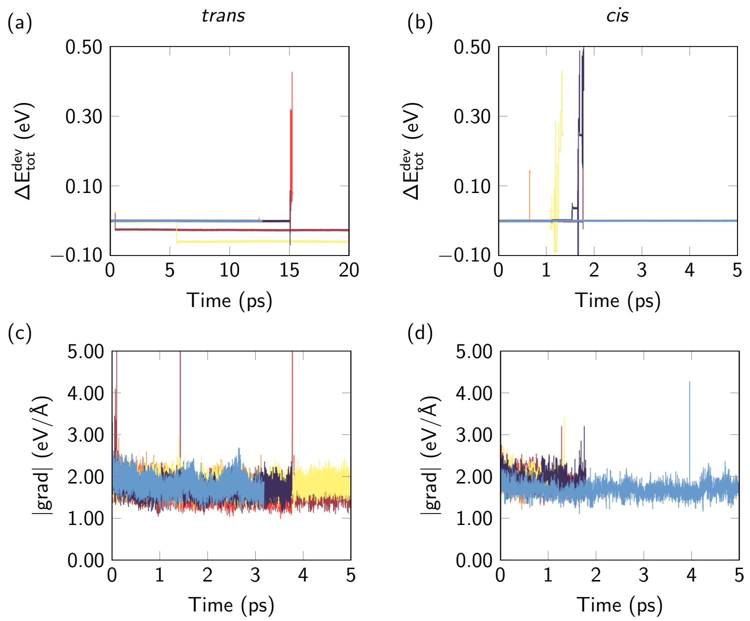

Figure S3: Total energy differences ($\Delta^{\rm dev}_{\rm tot} = E_{\rm tot}(t) - E_{\rm tot}(t_0)$) (a)-(b) and absolute gradient values (c)-(d) for individual trajectories.

Unphysical gradient spikes, and therefore sudden changes in the total energy were encountered in some of the dynamics trajectories (see Fig. S3), consistent with previously reported issues related to active/inactive orbital mixing in OM2 calculations.[173] When such artifacts occurred before a hop to the ground state, the affected trajectories were discarded from further analysis. If the artifacts occurred after the hop—*i.e.*, in the ground state—they were retained, since the ground-state geometries were excluded from the analysis regardless. In any case, trajectories in the ground state often crashed, as the open-shell Hartree–Fock is not reliable for ground-state dynamics.

Table S1: Vertical excitation energies of the first bright charge-transfer $\pi\pi^*$ state and the corresponding oscillator strengths for *trans*-DANS and *cis*-DANS adsorbed on different surfaces A—D[164] where the surface is represented as point charges, computed at the OM2/MRCI and TD-DFT/CAM-B3LYP level of theory. The RMSD of the excitation energies is 0.17 eV for the *trans* isomer and 0.18 eV the *cis* isomer.

	***trans*-DANS**					***cis*-DANS**			
	**OM2/MRCI**		**TDDFT**			**OM2/MRCI**		**TDDFT**	
Calc.	S_1	f_{osc}	S_1	f_{osc}	Calc.	S_1	f_{osc}	S_1	f_{osc}
A1	3.31	1.45	3.35	1.58	A7	3.28	0.79	3.29	0.70
A2	2.83	1.26	3.02	1.52	A8	2.55	0.54	2.77	0.57
A3	2.75	1.30	2.94	1.62	A9	2.72	0.69	2.87	0.71
A4	2.86	1.29	3.04	1.55	A10	2.39	0.35	2.62	0.38
A5	3.05	1.41	3.17	1.60	A11	3.35	0.56	3.36	0.47
A6	2.47	1.30	2.82	1.76	A12	2.77	0.42	2.86	0.42
					A13	2.84	0.75	2.97	0.75
					A14	3.22	0.62	3.25	0.55
B1	3.40	1.49	3.42	1.59	B7	2.99	0.69	3.08	0.67
B2	2.54	1.19	2.85	1.56	B8	2.62	0.68	2.83	0.72
B3	3.65	1.54	3.59	1.53	B9	2.51	0.36	2.71	0.38
B4	2.96	1.29	3.08	1.42	B10	2.29	1.11	2.61	0.42
B5	2.82	1.28	3.01	1.53	B11	3.58	0.71	3.56	0.50
B6	2.98	1.33	3.15	1.53	B12	2.72	0.49	2.85	0.50
					B13	2.40	0.57	2.65	0.63
					B14	2.75	1.23	3.14	0.51
C1	3.45	1.50	3.45	0.64	C7	3.19	0.72	3.22	0.67
C2	2.70	1.23	2.93	1.54	C8	2.15	0.51	2.51	0.63
C3	3.02	1.34	3.15	1.54	C9	2.80	0.39	2.90	0.36
C4	3.10	1.29	3.18	1.23	C10	2.62	0.61	2.81	0.64
C5	2.85	1.21	3.03	1.40	C11	3.30	0.78	3.30	0.69
C6	2.90	1.30	3.06	1.54	C12	2.80	0.64	2.92	0.65
					C13	3.44	0.66	3.41	0.51
					C14	2.73	0.70	2.90	0.72
D1	3.53	1.53	3.52	1.60	D7	3.13	0.74	3.18	0.70
D2	2.70	1.22	2.94	1.53	D8	2.84	0.58	2.94	0.61
D3	2.96	1.32	3.10	1.52	D9	3.37	0.70	3.34	0.57
D4	2.95	1.32	3.11	1.47	D10	2.85	0.60	2.96	0.60
D5	2.97	1.26	3.13	1.37	D11	3.60	0.64	3.56	0.50
D6	3.21	1.43	3.30	1.48	D12	3.13	0.46	3.16	0.41
					D13	3.56	0.71	3.52	0.46
					D14	2.81	0.53	2.93	0.52

S4 Static Scan of Excited-State Relaxation Pathways

Table S2: Energies in eV and oscillator strengths (f_{osc}) at the optimized geometries from Fig. 3. The states involved in the optimization are highlighted in bold.

Opt. geometry	Label	S_0 state	S_1 state	S_2 state	$f_{osc}^{S_1}$	$f_{osc}^{S_2}$
S_0^tmin	1	**0.00**	3.64	4.99	1.29	0.55
S_1^tmin	3	0.19	**3.30**	4.53	1.54	0.42
$TS^{t\text{-}iso}$	4	1.16	**3.63**	4.49	1.11	0.11
S_1/S_0^{iso}	5	**3.30**	**3.30**	6.44	0.00	0.01
$TS^{t\text{-}pyr}$	8	1.79	**3.82**	5.04	0.46	0.00
$S_1/S_0^{t\text{-}pyr}$	9	**3.49**	**3.49**	5.31	0.00	0.00
S_0^cmin	2	**0.13**	3.78	4.99	0.37	0.53
S_1^cmin	6	0.49	**3.25**	4.59	0.47	0.24
$TS^{c\text{-}iso}$	7	1.55	**3.53**	4.78	0.21	0.08
TS^{cyc}	10	1.27	**3.47**	4.40	0.61	0.06
S_1/S_0^{cyc}	11	**3.11**	**3.11**	3.73	0.00	0.00
$S_1/S_0^{rot\&cyc}$	12	**3.04**	**3.06**	5.90	0.00	0.03
$S_1/S_0^{pyr\&cyc}$	13	**2.97**	**2.97**	5.88	0.00	0.19

Table S3: Energy barriers located between S_1 minima and CIs obtained from calculation of minimal energy pathways, along with the energy gap between the ground state and S_1 state at the barrier. All the energies are given in eV.

	$S_1/S_0^{t\text{-}pyr}$ 9	*trans*-S_1/S_0^{iso} 5	*cis*-S_1/S_0^{iso} 5	S_1/S_0^{cyc} 11	$S_1/S_0^{rot\&cyc}$ 12	$S_1/S_0^{pyr\&cyc}$ 13
	From static scan					
$\Delta E_{S_1-S_0}$ gap	2.03	2.47	2.20	1.98	0.65	0.84
Barrier in the S_1	0.53	0.33	0.28	0.22	0.43	0.52

Figure S4 shows the three additional non-isomerization pathways of the *cis* isomer. These pathways are also included in the main manuscript (see Figure 3), while here we additionally present the corresponding minimum-energy pathways. Although the optimizations toward the two CIs, $S_1/S_0^{rot\&cyc}$ and $S_1/S_0^{pyr\&cyc}$, did not converge, we believe that these results still provide valuable information. Nonadiabatic dynamics simulations and excited-state umbrella sampling revealed these additional relaxation pathways towards $S_1/S_0^{rot\&cyc}$ and $S_1/S_0^{pyr\&cyc}$ (two last panel) characterized by the rotation/twisting or pyramidalization of the NO_2 functional group. These pathways occur more frequently than the traditional stilbene-type cyclization (first panel in Figure S4). Based on the energy barriers presented in Table S3, the calculated barrier to access this new cyclization pathway for the *cis* isomer appears significantly higher (0.43 eV and 0.52 eV) than those for stilbene-like cyclization or isomerization. However, for the two CIs associated with NO_2 rotation and pyramidalization, a TS could not be fully optimized. Potential energy scans obtained from unconverged nudged elastic band calculation suggest a region where the S_1-S_0 energy gap becomes small but non-zero, providing an alternative kinetic route to the CI. These findings underscore that optimized static barriers do not always accurately mirror the actual branching ratios observed in nonadiabatic dynamics.

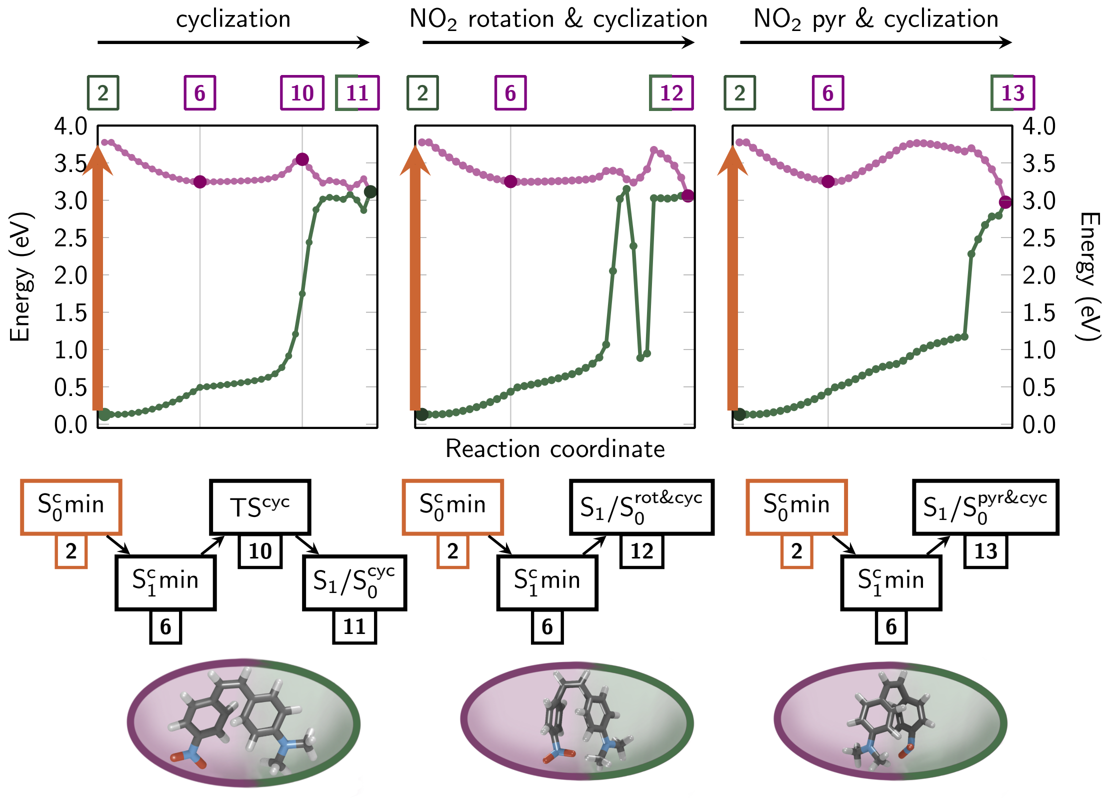

Figure S4: Alternative relaxation pathways of *cis*-DANS optimized at the OM2/MRCI level of theory. Critical points along the pathway are shown by vertical light gray lines and numbers above them. S_1 energies are represented in light purple, while S_0 energies are shown in green. The orange color (arrow and rectangles) denotes excitation at the Franck-Condon region. Numbers above the graph correspond to critical points and indicate when different paths pass through the exact same geometries. Below the graph, geometries of MECIs for the relevant path are shown.

S5 Excited-state population fitting

In Figure 4 of the main text, the electronic populations were fitted using a sequential first-order model with three components and two time constants to introduce a "natural" delay in the fitting scheme instead using an artificial constant delay term, as has been done in the literature.[132,193–195] Here we show how a delay emerges naturally from the model and derive the analytic form of the build-up (delay).

A sequential first-order scheme:

$$A_0 \xrightarrow{k_1} A_1 \xrightarrow{k_2} \dots \xrightarrow{k_M} A_M. \tag{31}$$

Assuming that at $t = 0$ all population is in A_0 ($A_0(0) = 1, A_{r>0} = 0$), the rate equations are:

$$\frac{\mathrm{d}A_0}{\mathrm{d}t} = -k_1 A_0, \qquad r = 0, \tag{32}$$

$$\frac{\mathrm{d}A_r}{\mathrm{d}t} = +k_r A_{r-1} - k_{r+1} A_r, \qquad r = 1, \dots, M-1, \tag{33}$$

$$\frac{\mathrm{d}A_M}{\mathrm{d}t} = +k_M A_{M-1} \qquad r = M; \tag{34}$$

If we assume equal rates $k_1 = k_2 = \cdots = k_M$, the solution can be written as:

$$A_r(t) = \frac{(kt)^r}{r!}\mathrm{e}^{-kt}, \qquad r = 0, \dots, M-1. \tag{35}$$

This analytical solution corresponds mathematically to the probability density function of the Erlang distribution. The population of the first $M - 1$ intermediates, the part of the system that must be depleted before A_M can build-up, is therefore

$$1 - A_M(t) = a_M(t) = \sum_{r=0}^{M-1} A_r(t) = \mathrm{e}^{-kt} \sum_{r=0}^{M-1} \frac{(kt)^r}{r!}. \tag{36}$$

To estimate the delay time, we calculate the inflection point

$$\frac{\mathrm{d}^2}{\mathrm{d}t^2} a_M(t) = 0. \tag{37}$$

To keep the derivation simple, we introduce a new constant $\eta = kt$ and define

$$S_M(\eta) = \sum_{m=0}^{M-1} \frac{\eta^m}{m!}, \tag{38}$$

therefore $a_M(t)$ take the following from $a_M(t) = \mathrm{e}^{-\eta} S_{M-1}(\eta)$. All derivatives may now be taken with respect to η.

$$\frac{\mathrm{d}}{\mathrm{d}\eta} a_M(t) = \frac{\mathrm{d}}{\mathrm{d}\eta}\left[\mathrm{e}^{-\eta}\right] S_M(\eta) + \mathrm{e}^{-\eta} \frac{\mathrm{d}}{\mathrm{d}\eta} S_M(\eta) \tag{39}$$

where the derivative of the second term is

$$\frac{\mathrm{d}}{\mathrm{d}\eta} S_M(\eta) = \sum_{m=1}^{M-1} \frac{m\eta^{m-1}}{m!} = \sum_{m=1}^{M-1} \frac{\eta^{m-1}}{(m-1)!} = \sum_{m=0}^{M-2} \frac{\eta^m}{m!} = S_{M-1}(\eta) \tag{40}$$

therefore the first derivative can be written

$$\frac{\mathrm{d}}{\mathrm{d}\eta}a_M(t) = (-\mathrm{e}^{-\eta})S_M(\eta) + \mathrm{e}^{-\eta}S_{M-1}(\eta)$$
$$= \mathrm{e}^{-\eta}\Big[S_{M-1}(\eta) - S_M(\eta)\Big] \tag{41}$$

and

$$\frac{\mathrm{d}}{\mathrm{d}t}a_M(t) = \frac{\mathrm{d}\eta}{\mathrm{d}t}\frac{\mathrm{d}}{\mathrm{d}\eta}a_M(t) = k\ \mathrm{e}^{-\eta}\left[S_{M-1}(\eta) - S_M(\eta)\right]\ . \tag{42}$$

We differentiate once more, again with the product rule:

$$\frac{\mathrm{d}^2}{\mathrm{d}t^2}a_M(t) = k^2\left(\frac{\mathrm{d}}{\mathrm{d}\eta}\Big[\mathrm{e}^{-\eta}\Big]\Big[S_{M-1}(\eta) - S_M(\eta)\Big] + \mathrm{e}^{-\eta}\frac{\mathrm{d}}{\mathrm{d}\eta}\Big[S_{M-1}(\eta) - S_M(\eta)\Big]\right)$$
$$= k^2\ \mathrm{e}^{-\eta}\Big[-\big(S_{M-1}(\eta) - S_M(\eta)\big) + S_{M-2}(\eta) - S_{M-1}(\eta)\Big] \tag{43}$$
$$= k^2\ \mathrm{e}^{-\eta}\Big[S_M(\eta) + S_{M-2}(\eta) - 2S_{M-1}(\eta)\Big].$$

In the second derivative every power of η cancels out except the highest ones ($m = M-2$ and $m = M-1$). So here we collect the terms that survive:

$$\Big[S_M(\eta) + S_{M-2}(\eta) - 2S_{M-1}(\eta)\Big] = \frac{\eta^{M-1}}{(M-1)!} - \frac{\eta^{M-2}}{(M-2)!} = \frac{\eta^{M-2}\big[\eta - (M-1)\big]}{(M-1)!}, \tag{44}$$

which we can insert back into equation (43)

$$\frac{\mathrm{d}^2}{\mathrm{d}t^2}a_M(t) = k^2\ \mathrm{e}^{-kt}\frac{(kt)^{M-2}\big[kt - (M-1)\big]}{(M-1)!}. \tag{45}$$

Equation (45) is zero when the bracket is zero:

$$kt = M - 1 \Rightarrow t = \frac{M-1}{k}. \tag{46}$$

For a linear, irreversible sequential first-order model $A_0 \xrightarrow{k_1} A_1 \xrightarrow{k_2} \dots \xrightarrow{k_M} A_M$ with equal first-order rate k, the combined population of the intermediates has an inflection point at $t = \frac{M-1}{k}$ (the value at that inflection point is $2e^{-1} \approx 0.74$ for $M = 2$ and converges to 0.5 for large M). Because every additional intermediate kinetic species contributes the same average waiting time $\frac{1}{k}$, adding one additional intermediate shifts the observable signal later by $\frac{1}{k}$. Thus the model supplies a built-in delay and no ad-hoc time-zero offset is needed to be introduced.

S6 Decorrelation Timescales

To ensure that the equilibration along the CV happens more rapidly than the electronic relaxation, we computed the autocorrelation function $C(t)$ of the CV

$$C(t) = \frac{\mathrm{Cov}(\xi_{\mathrm{i}}, \xi_{\mathrm{i+t}})}{\mathrm{Var}(\xi)} \tag{47}$$

for every single umbrella simulation and fitted it with a single decaying exponential function to obtain the decorrelation time τ

$$C(t) \approx e^{-t/\tau} \ . \tag{48}$$

The results for the different centers of umbrella simulations are summarized in Table S4.

Table S4: Number of umbrella windows as well as mean and standard deviation of the decorrelation times in fs for each umbrella window center.

Window Center / eV	Num. Simulations	$\langle\tau\rangle$ / fs	std(τ) / fs
0.0	1	21.6	0.0
0.1	1	20.8	0.0
0.2	1	21.2	0.0
0.3	1	15.8	0.0
0.4	1	66.8	0.0
0.5	1	12.8	0.0
0.6	7	9.0	6.0
0.7	7	6.2	2.5
0.8	7	6.4	2.4
0.9	7	9.6	4.7
1.0	7	6.6	3.0
1.1	7	5.9	4.3
1.2	7	6.2	3.1
1.3	7	6.1	6.4
1.4	7	5.3	2.1
1.5	7	5.8	1.4
1.6	7	5.9	2.7
1.7	7	6.6	2.0
1.8	7	9.1	3.8
1.9	7	7.3	5.6
2.0	7	7.6	5.0
2.1	7	7.3	4.7
2.2	7	7.2	6.8
2.3	7	17.5	16.3
2.4	7	19.4	9.5
2.5	7	9.1	4.4
2.6	7	12.1	3.5
2.7	7	21.5	4.2
2.8	7	28.4	11.4
2.9	7	32.3	6.2
3.0	7	27.3	9.0
3.1	7	25.3	8.8
3.2	7	21.5	5.3
3.3	7	22.3	8.6
3.4	7	20.0	3.5
3.5	7	19.6	4.3
3.6	7	21.3	10.3

S7 Nonadiabatic dynamics and excited-state umbrella sampling

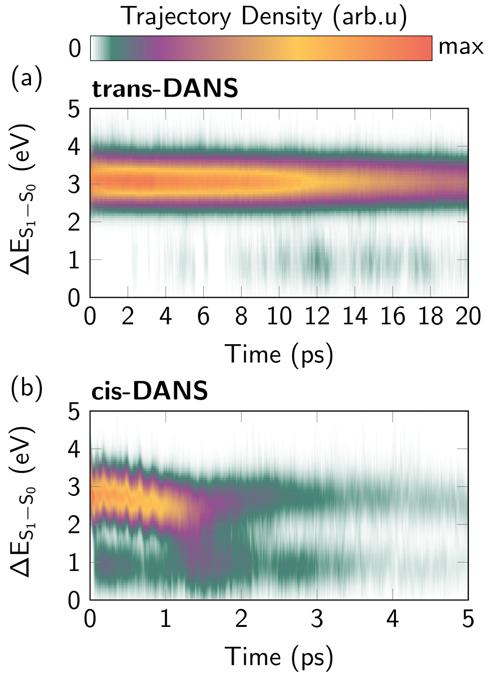

Figure S5: Gaussian convolution of S_1/S_0 energy gap for *trans* (a) and *cis* (b) isomers.

Figure S5 shows the Gaussian convolution of S_1/S_0 energy gap obtained from the unbiased simulations, proving the existence of meta stable regions for both, the *trans* and *cis* isomer, around 1 eV, matching the positions identified in the free-energy profiles in Figure 7.

Here, we compare the free-energy and PMF profiles obtained from excited-state umbrella sampling simulations, using either the S_1/S_0 energy gap and the HCCH dihedral angle as the CV for both the *trans* and *cis* isomers. In both cases, the resulting PMF and free-energy profiles are very similar. However, when using the HCCH dihedral angle as the CV, the two curves remain basically parallel throughout, whereas with the energy gap as the CV, the curves exhibit small deviations at certain points. This illustrates that the PMF can be influenced by the choice of the CV, highlighting the importance of transforming the CV into a coordinate system that minimizes this dependence. Nevertheless, as previously mentioned, even with the energy gap as the CV, the observed differences are relatively small.

However, the energy profiles from HCCH-constrained sampling in Figure S6 are incomplete for the *trans* isomer, with the 180° region missing. Key features near the CI, such as the sharp increase in free energy observed in Figure 7 cannot be captured for neither of the isomers, because the projection onto the dihedral angle includes high and low energy gap geometries for every value of the angle. Or in other words, there is no one-to-one mapping between the dihedral angle and the energy gap such that frames with $\phi_{\mathrm{HCCH}} = 90°$ do not immediately correspond to configurations at the CI seam.

In Figure S7, free-energy profiles are shown for two sampling approaches: one using the S_1/S_0 energy gap as the CV and one using the dihedral angle of the central carbon atoms HCCH as the CV. Both profiles exhibit a similar shape and display the characteristic increase in free energy near the

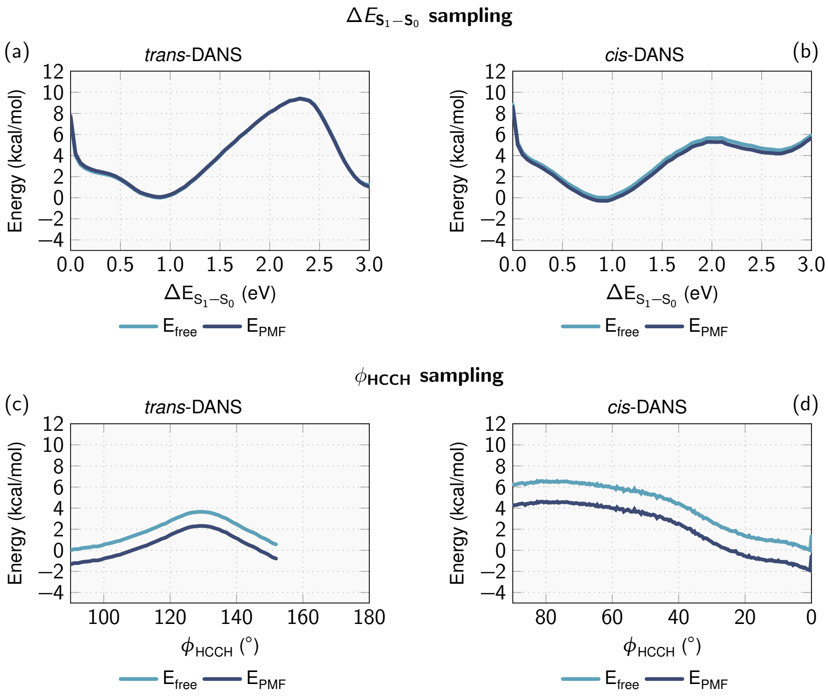

Figure S6: Comparison of PMF and free-energy profiles obtained from umbrella sampling using the S_1/S_0 energy gap as CV (panel (a) for *trans* and panel (b) for *cis* isomer) and the HCCH dihedral angle as CV (panel (c) for *trans* and panel (d) for *cis* isomer).

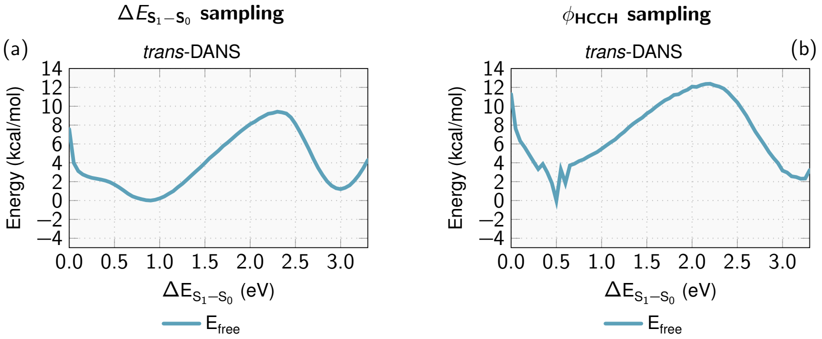

Figure S7: Free-energy profiles projected onto the energy gap S_1/S_0 coordinate, obtained by sampling with (a) the energy gap $\Delta E_{S_1-S_0}$, and (b) the dihedral angle ϕ_{HCCH}.

CI. Sampling with the dihedral angle, however, results in more geometries located in higher-energy regions, which leads to a higher apparent barrier. In contrast, using the energy gap as the CV guides the sampling through energetically more accessible regions.

S8 Energy Gap Distribution At Hop

Figure S8 shows the frequency of hops to the ground state at different S_1–S_0 energy gaps. A substantial number of hops occur at energy gaps greater than 0.5 eV, indicating that the presence of a second minimum (see in Figure 7a-b and d-e) around 1 eV plays a crucial role. This minimum allows the molecule to become temporarily trapped and stabilized, increasing the likelihood of transition to the ground state before reaching the CI region, where the free energy rises sharply and the entropy drops significantly hence the molecule is less likely to be there for longer time.

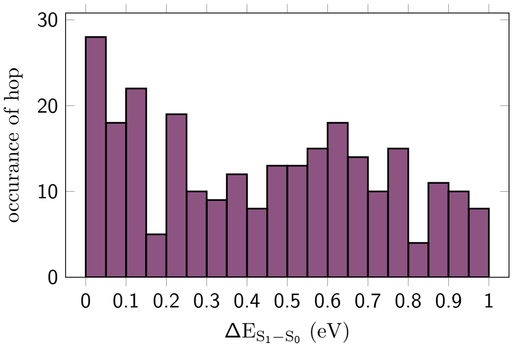

Figure S8: Distribution of nonadiabatic hops at different energy gaps $\Delta E_{S_1-S_0}$ for both isomers, obtained from MRCI/OM2 nonadiabatic dynamics.

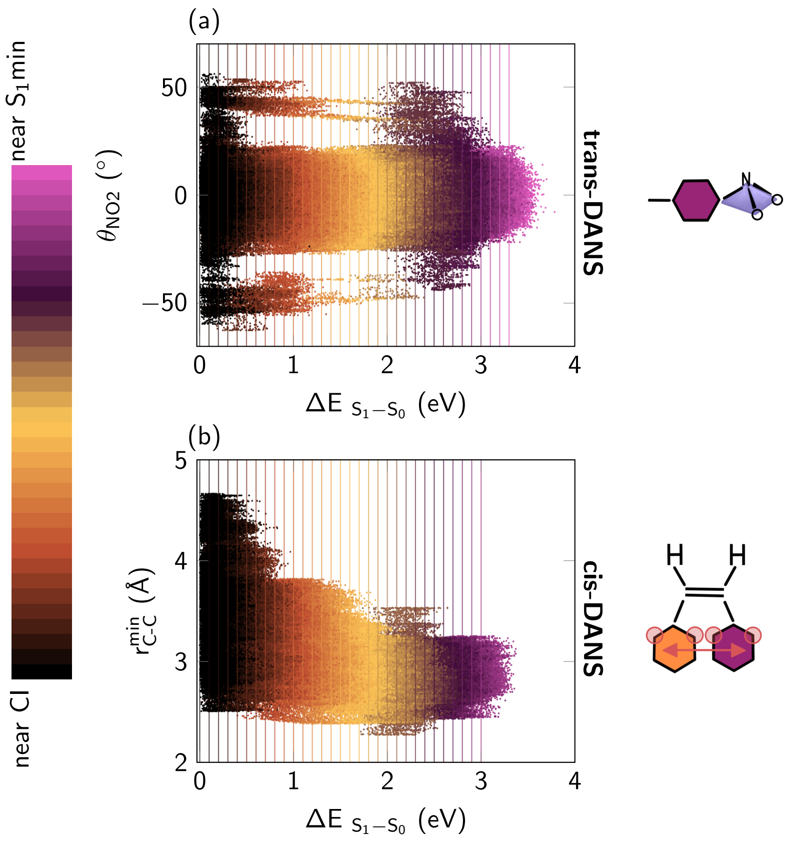

Figure S9: Projection of the simulation frames onto the NO_2 pyramidalization angle θ_{NO2} and the S_1/S_0 energy gap for *trans*-DANS (a) and onto the minimum distance out of four carbon atoms on opposite rings $r^{min}_{C\text{-}C}$ of *cis*-DANS (b). The results were obtained using the energy gap $\Delta E_{S_1-S_0}$ as CV. Vertical and horizontal lines indicate the centers of the applied bias potentials.

For the umbrella sampling calculations using the S_1-S_0 energy gap as the CV, additional geometric parameters were also monitored shown in Figure S9. These are the same types of geometric descriptors discussed for the nonadiabatic dynamics in Figure 6. For the *trans* isomer, the NO_2 pyramidalization angles were collected from the different sampling windows. In Figure S9a, the large pyramidalization characteristic of the CI region (around -50°—50°) is reached mainly at small S_1-S_0 energy gaps below 1 eV. Larger pyramidalization angles are generally rarely observed at higher energy gaps. This indicates that the pathway toward the pyramidalized CI is characterized by small pyramidalization angles over most of the relaxation process, with stronger pyramidalization becoming typical only at very small energy gaps. Consequently, when the pyramidalization angle is plotted against the S_1-S_0 energy gap, the two pathways overlap much more strongly than in the case of the HCCH dihedral angle shown in Figure 8.

In Figure S9b, the minimum distance between the two opposite carbon atoms of the rings, as indicated in the schematic figure, is shown. Up to an S_1-S_0 energy gap of about 1.5 eV, the distributions largely overlap. Below this value, however, two trends become visible: one pathway leads to larger minimal distances, while the other remains at a similar distance, in analogy to the behavior observed in the nonadiabatic dynamics in Figure 6. However, distances around 2 Å are not observed, indicating that the stilbene-like cyclization pathway was not sampled.
